# Supplementary material for: Charge redistribution dynamics in chalcogenide-stabilized cuprous electrocatalysts unleash ampere-scale partial current toward formate production
Source: Nat Commun. 2025 Oct 24;16:9426. doi: 10.1038/s41467-025-64472-1 (PMC12552683; doi:10.1038/s41467-025-64472-1)
Supplement: Supplementary file 1 — Supplementary Information [file 41467_2025_64472_MOESM1_ESM.pdf]

Supplementary Information

Charge redistribution dynamics in chalcogenide-stabilized cuprous electrocatalysts unleash ampere-scale partial current toward formate production

Feng-Ze Tian[1], Wen-Jui Chang[1], Pei-Jung Liang[1], Yi-An Lai[1], Chia-Shuo Hsu[2], Sheng-Chih Lin[1], Yu-Hsin Chen[3], You-Chiuan Chu[1], Shih-Wen Huang[4], Hui-Lung Chen[5*], Hao Ming Chen[1,2,3,6*]*

[1]Department of Chemistry, National Taiwan University, Taipei, 10617, Taiwan

[2]National Synchrotron Radiation Research Center, Hsinchu, 30076, Taiwan

[3]Graduate School of Advanced Technology, National Taiwan University, Taipei, 10617, Taiwan

[4]PSI Center for Photon Science, Paul Scherrer Institute, Forschungsstrasse 111, 5232 Villigen, Switzerland

[5]Department of Chemical and Materials Engineering, Chinese Cultural University, Taipei, 111, Taiwan

[6]Center for Emerging Materials and Advanced Devices, National Taiwan University, Taipei 10617, Taiwan

*Corresponding author.

Email address: haomingchen@ntu.edu.tw (H. M. Chen); chl3@ulive.pccu.edu.tw (H-L. Chen); shih.huang@psi.ch (S-W. Huang)

Supplementary Figures

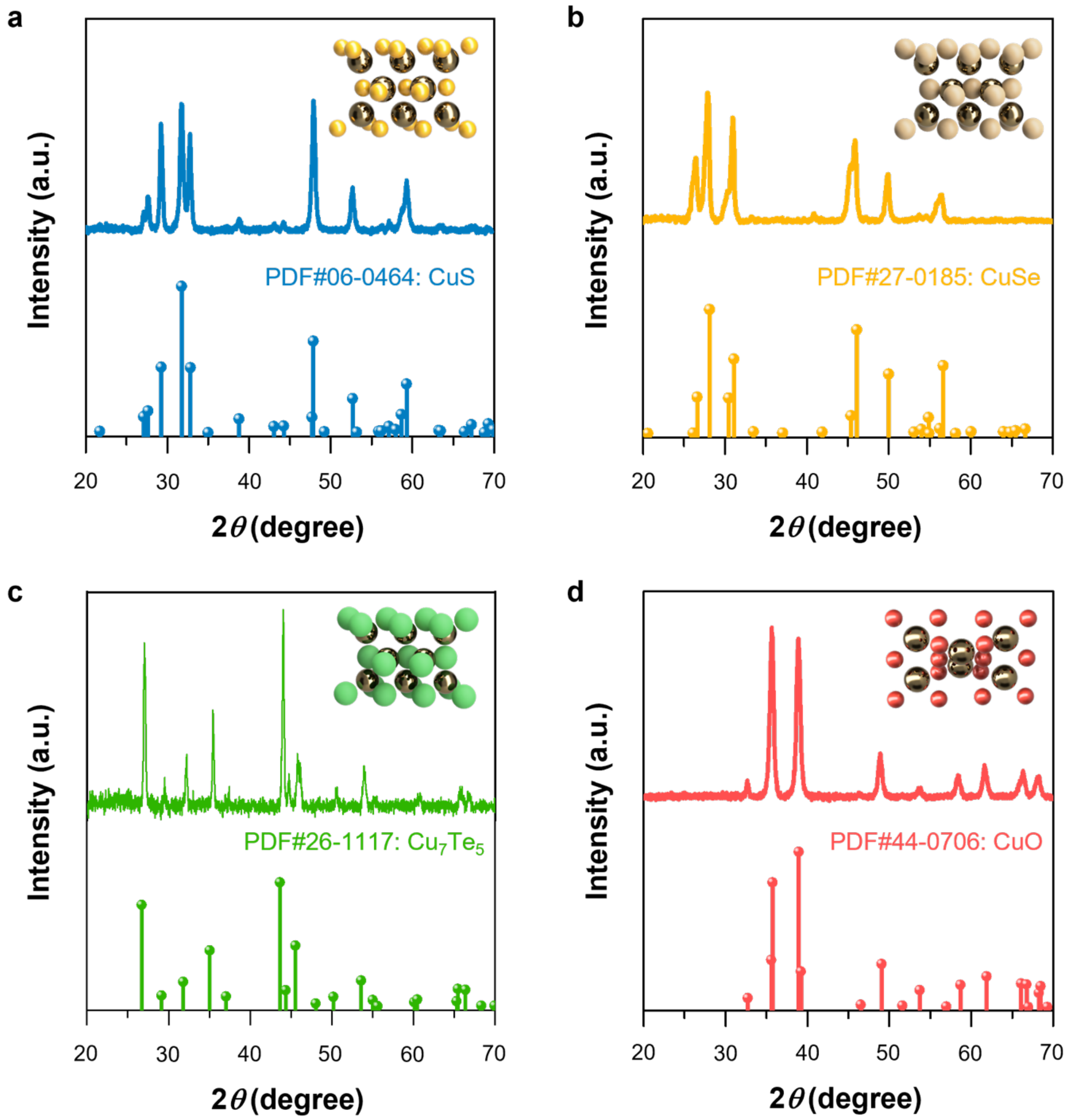

Supplementary Fig. 1. X-ray diffraction (XRD) patterns of **a,** CuS, **b,** CuSe, **c,** CuTe, and **d,** CuO. Source data are provided as a Source Data file.

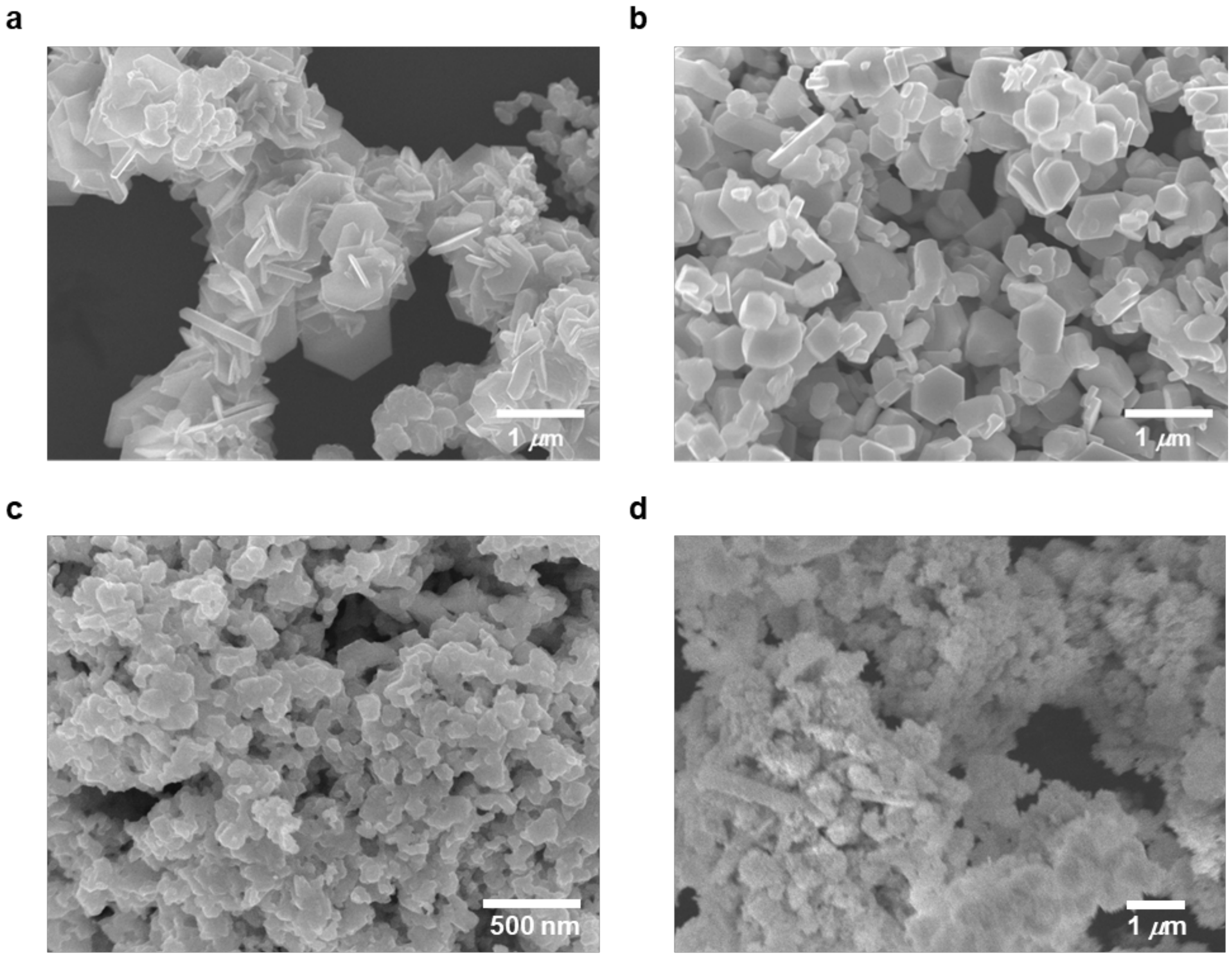

Supplementary Fig. 2. Typical SEM images of **a,** CuS, **b,** CuSe, **c,** CuTe, and **d,** CuO.

Supplementary Note 1

Due to the structural difference in Rickardite (Cu_7Te_5) and Vulcanite (CuTe), the oxidation state of the as-prepared CuTe might be less positive than that of the original Vulcanite (CuTe) as expected. This suggestion could also be further supported by the XANES spectra in Supplementary Fig. 3 and Supplementary Table 2, the spectra show that the absorption edge position of the CuTe was less positive than those of CuS and CuSe, indicating a slightly lower oxidation state on the CuTe. Notes that an additional peak appears at 2.24 Å in the CuTe R-space spectrum (Supplementary Fig. 4), which could be attributed to the contribution of Cu−Cu coordination that have substituted the original Te site in Vulcanite (CuTe) crystal phase to form Cu_7Te_5. The quantitative analyses reveal two similar interatomic distances (2.58 Å, 2.58 Å) and coordination numbers (1.9, 2.2) for Cu−Cu and Cu−Te pairs (detailed fitting parameters are summarized in Supplementary Table 3).

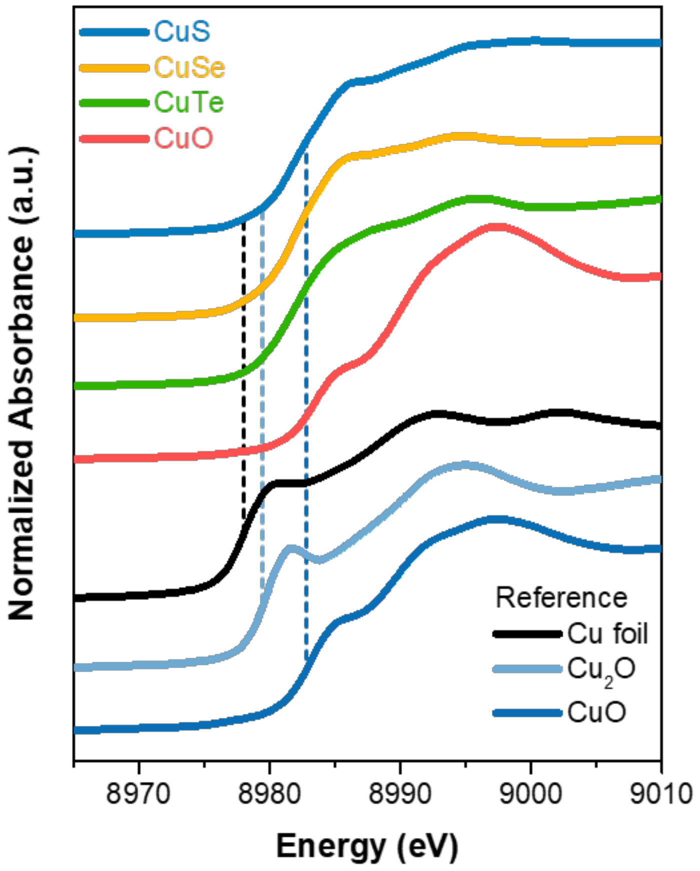

Supplementary Fig. 3. XANES spectra of Cu K-edge for as-prepared catalysts and corresponding references. Source data are provided as a Source Data file.

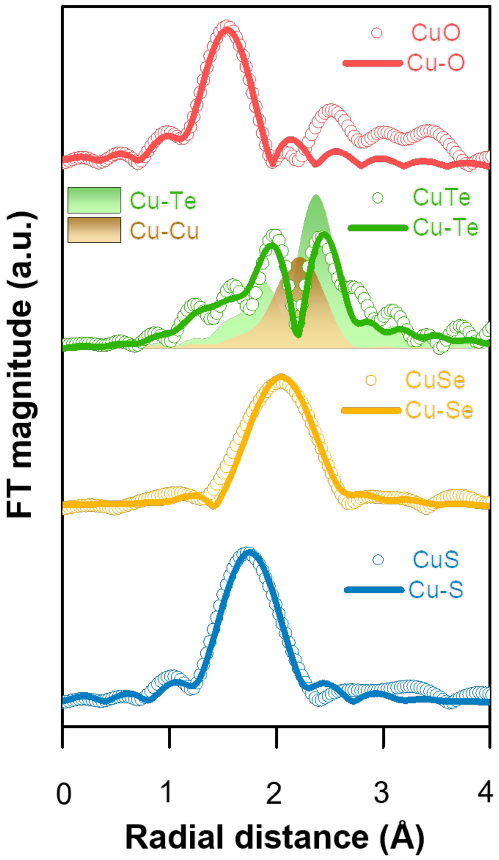

Supplementary Fig. 4. k^2-weighted FT-EXAFS of Cu K-edge for as-prepared catalysts. Source data are provided as a Source Data file.

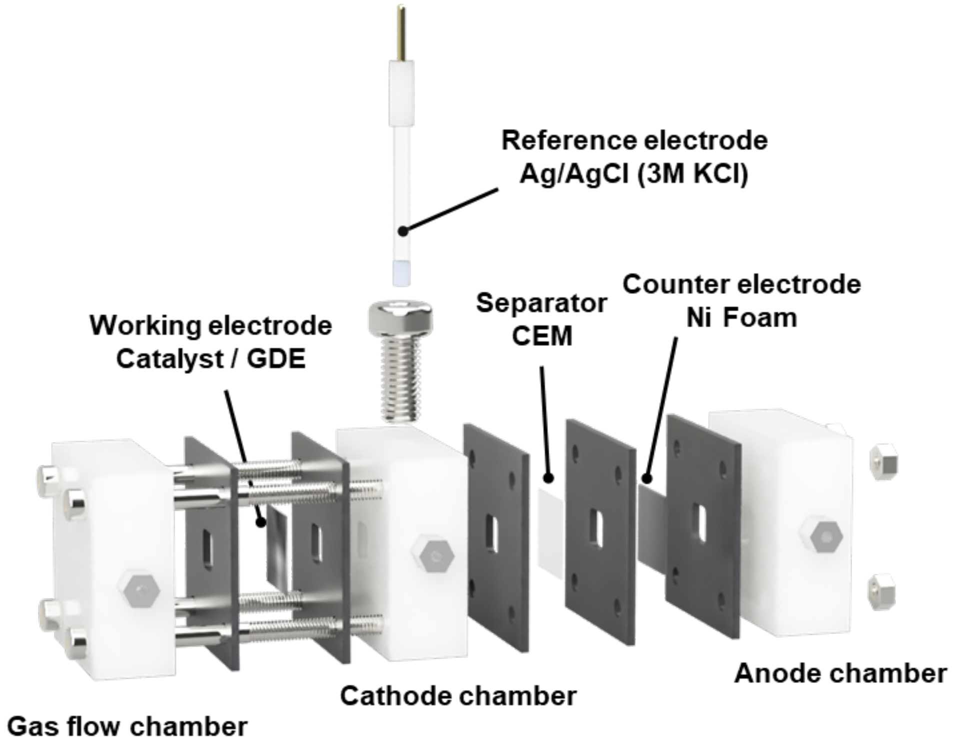

Supplementary Fig. 5. Schematic representation of gas diffusion flow cell, where CEM stands for cation exchanged membrane, and GDE is gas diffusion electrode.

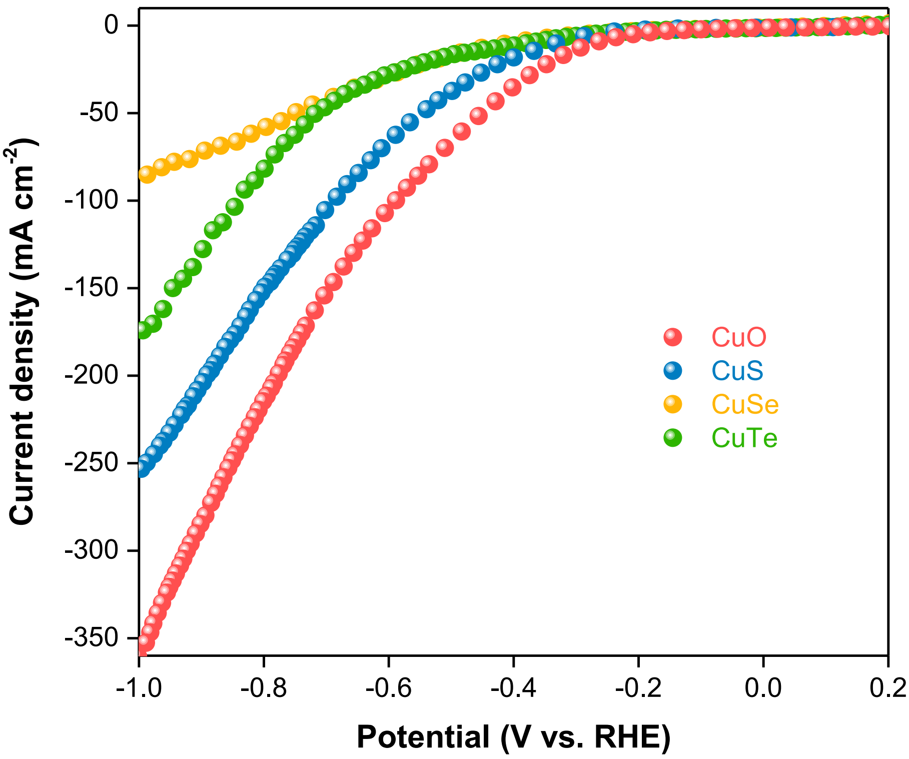

Supplementary Fig. 6. Linear sweep voltammetry (LSV) polarization curves at scan rate of 100 mV s^{-1} in 1.0 M KOH with a CO_2 flow rate of 30 sccm. All potentials are reported versus RHE with 85% *iR*-correction (*i*, current; *R*, solution resistance). Source data are provided as a Source Data file.

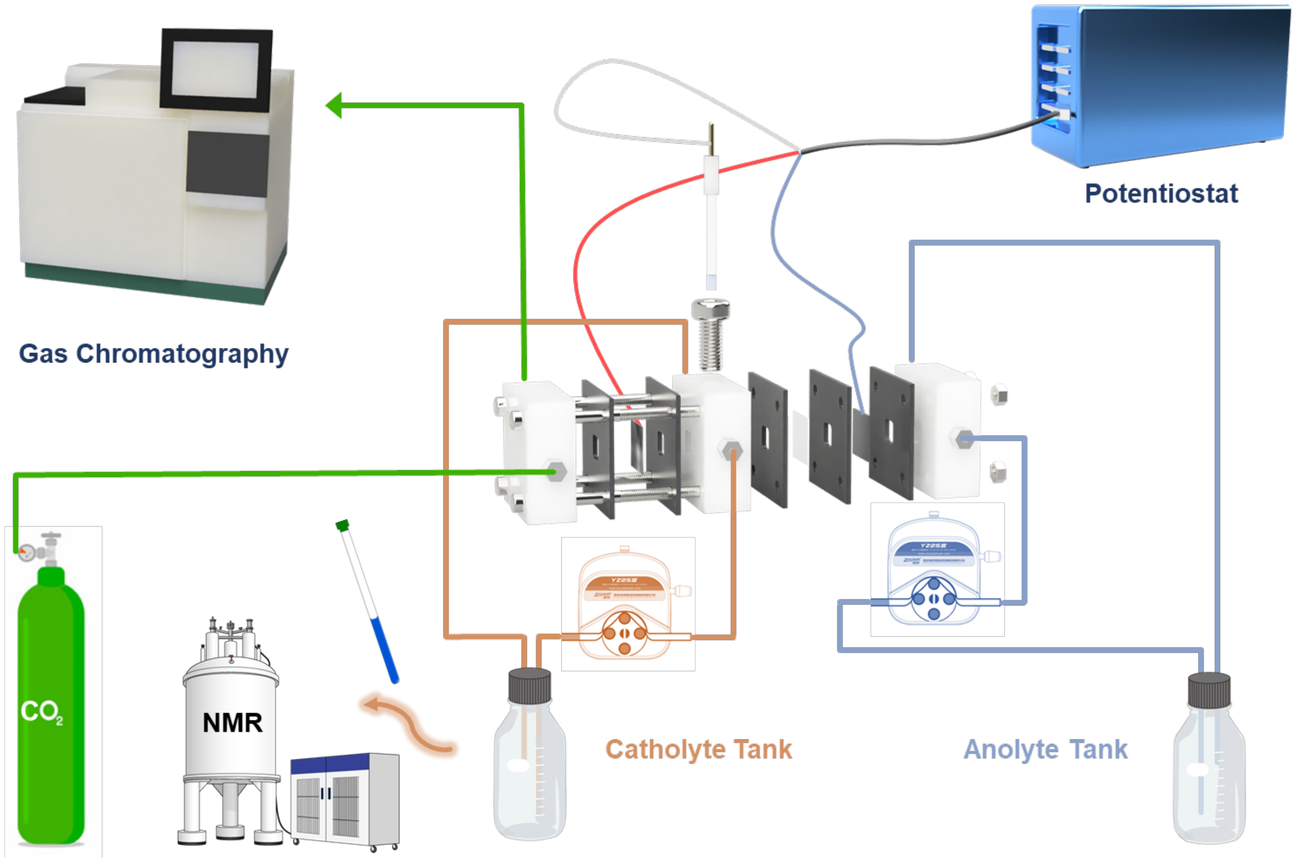

Supplementary Fig. 7. Experimental setup for evaluating CO_2RR product efficiency.

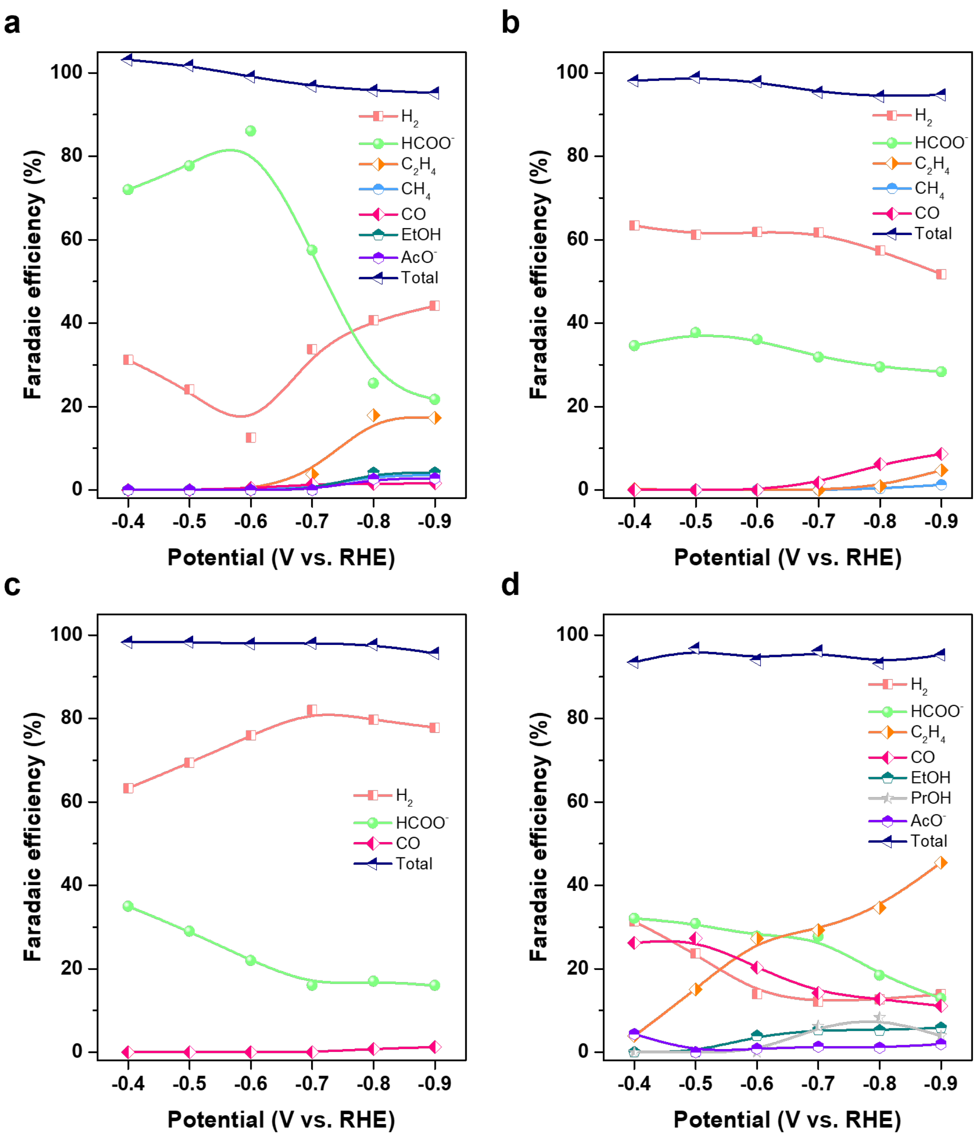

Supplementary Fig. 8. Faradaic efficiency as a function of potential of **a,** CuS, **b,** CuSe, **c,** CuTe, and **d,** CuO. All potentials are reported versus RHE with 85% *iR*-correction (*i*, current; *R*, solution resistance). Source data are provided as a Source Data file.

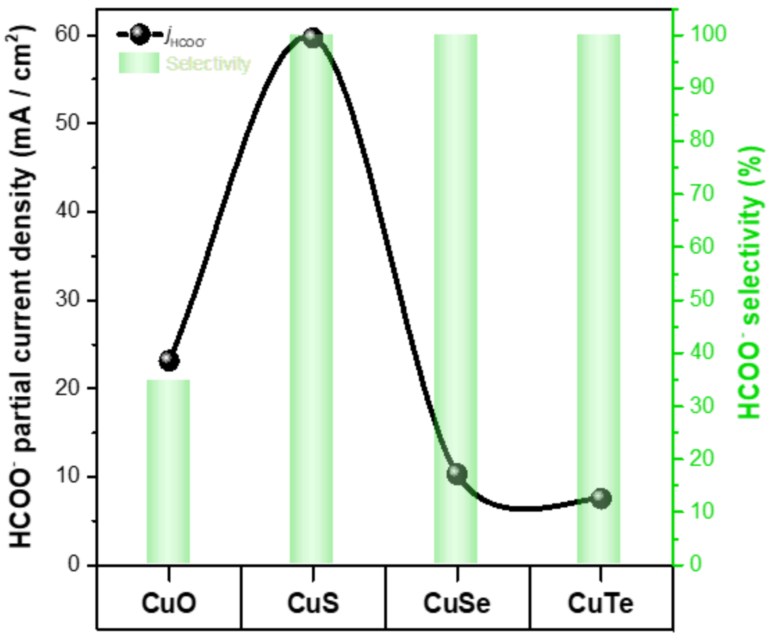

Supplementary Fig. 9. Partial current density and selectivity of formate at –0.6 V for CuO, CuS, CuSe, and CuTe. Source data are provided as a Source Data file.

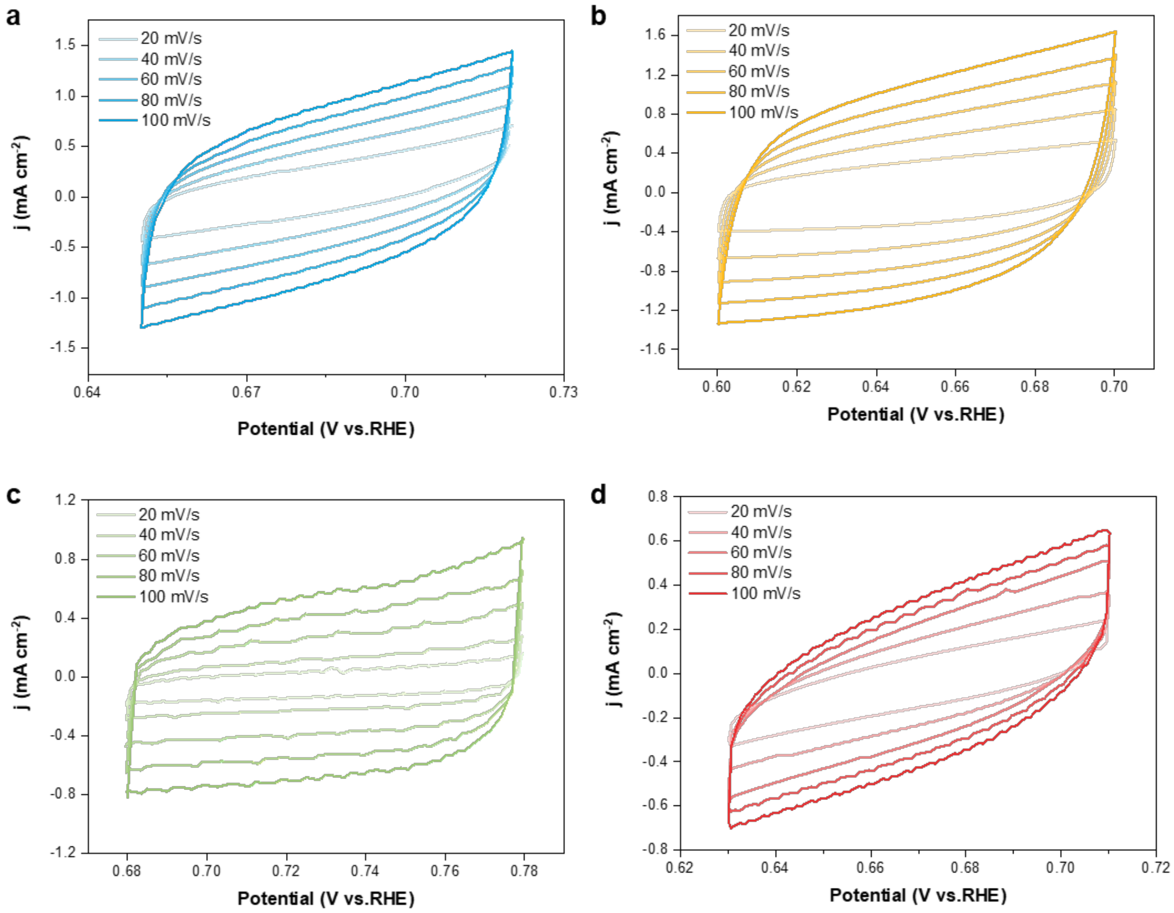

Supplementary Fig. 10. CV curves with various scanning rate of **a,** CuS **b,** CuSe **c,** CuTe, and **d,** CuO. Source data are provided as a Source Data file.

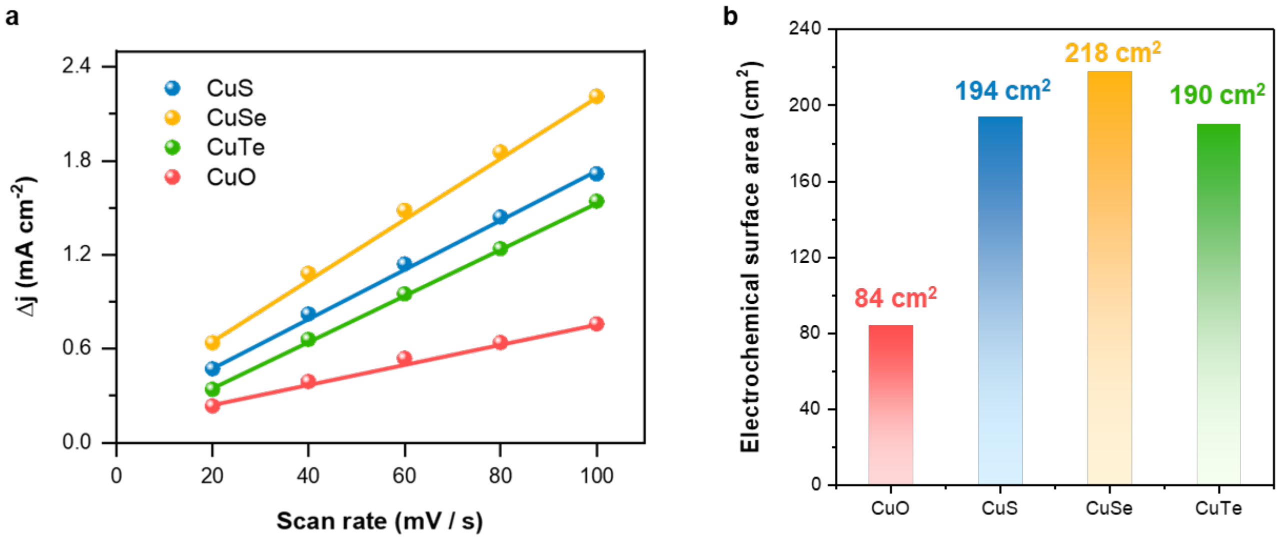

Supplementary Fig. 11. Electrochemically active surface area (ECSA) analysis based on double-layer capacitance. **a,** linear plot of current density as a function of scan rate, and **b,** ECSA value for different catalysts. The corresponding double-layer capacitance (C_{dl}) was determined as the half of slope. ECSA for different electrocatalysts could be extracted based on double-layer capacitance. Source data are provided as a Source Data file.

Supplementary Note 2

Due to design limitations of the current SEIRAS cell, which does not accommodate a gas diffusion electrode (GDE), it is not feasible to spatially separate the CO_2 gas stream from the alkaline electrolyte. Consequently, the SEIRAS measurements were conducted by purging CO_2 into a CO_2-pre-saturated 1.0 M KOH solution. Although this inevitably results in the conversion of KOH to $KHCO_3$ during the course of the experiment, this configuration was deliberately chosen to closely replicate the reaction environment employed in our electrochemical performance evaluations, which utilized continuous CO_2 flow with1.0 M KOH electrolyte. Importantly, the vibrational signatures of key surface-bound intermediates detected via SEIRAS are consistent with those observed in the *in situ* Raman experiments conducted under well-controlled flow conditions. While this SEIRAS configuration does not represent a fully ideal operando setup for alkaline CO_2RR, it still provides reliable and relevant mechanistic insights.

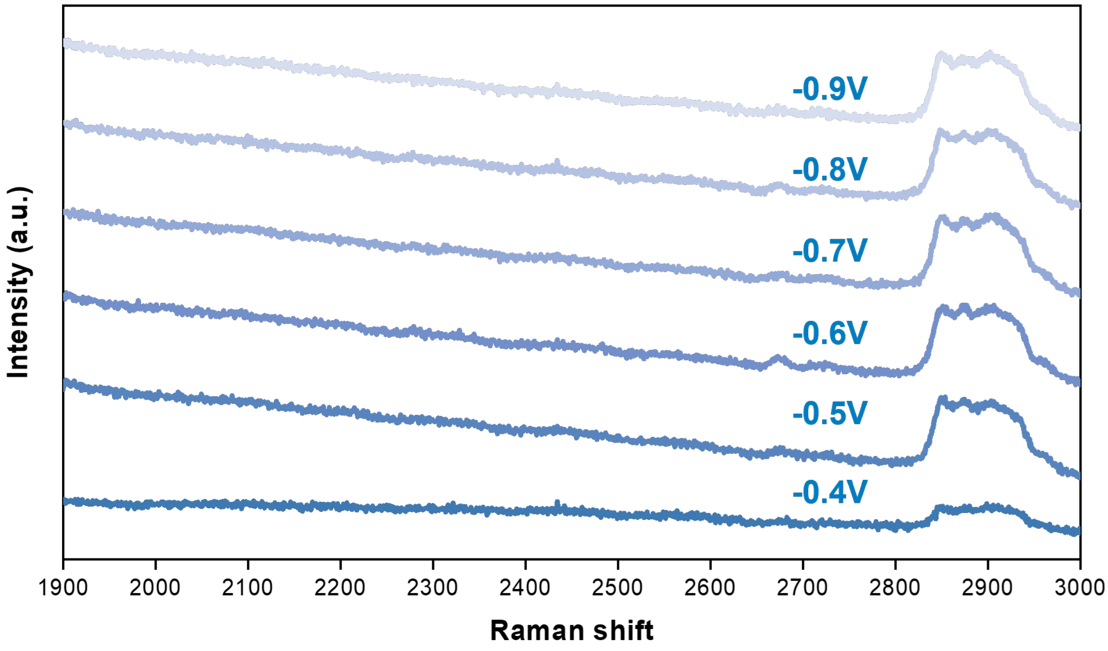

Supplementary Fig. 12. *In situ* Raman spectra as a function of potential for CuS. All potentials are reported versus RHE with 85% *iR*-correction (*i*, current; *R*, solution resistance). Source data are provided as a Source Data file.

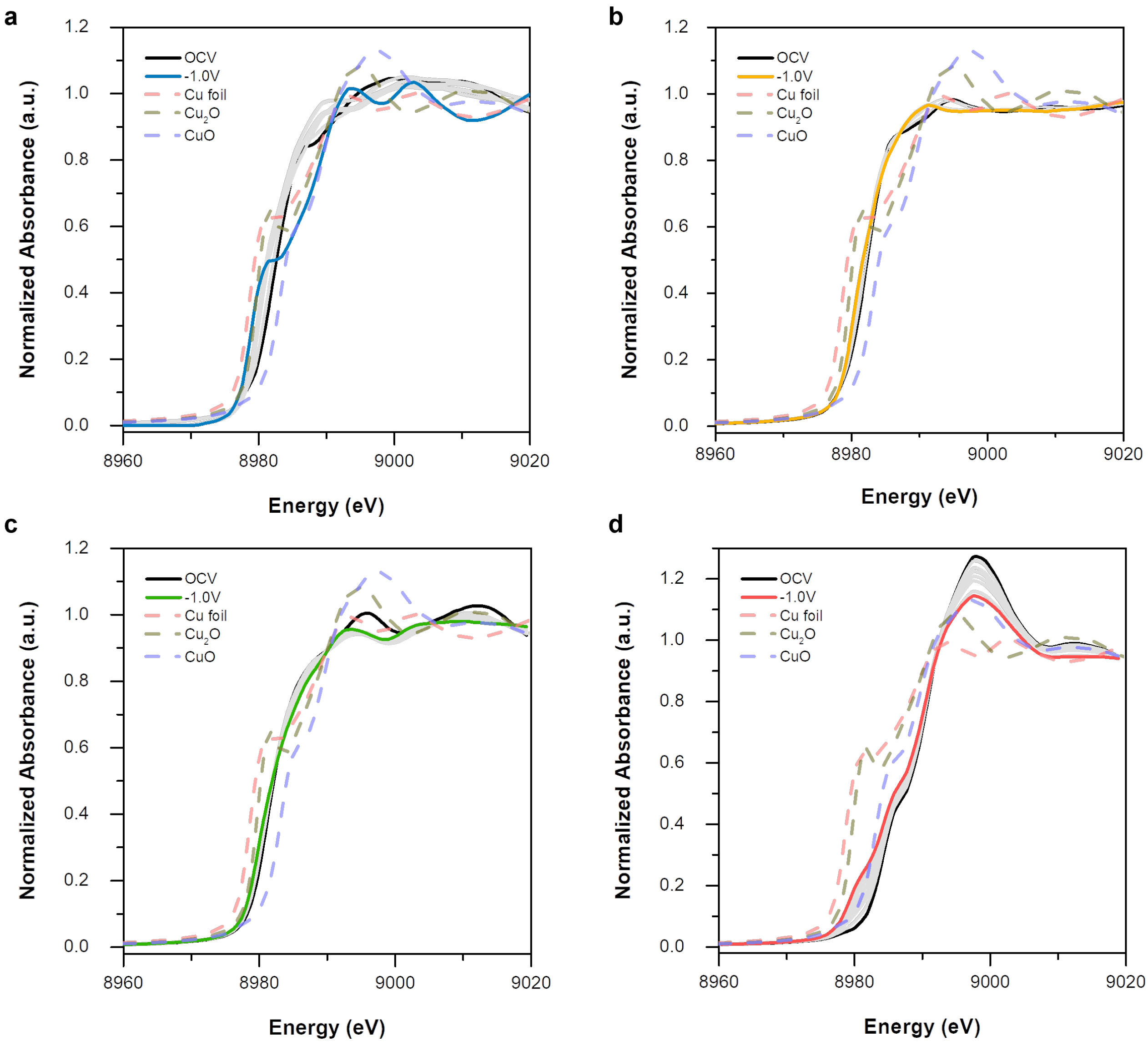

Supplementary Fig. 13. *In situ* Cu K-edge XANES of **a,** CuS, **b,** CuSe, **c,** CuTe, and **d,** CuO. All potentials are reported versus RHE with 85% *iR*-correction (*i*, current; *R*, solution resistance). Source data are provided as a Source Data file.

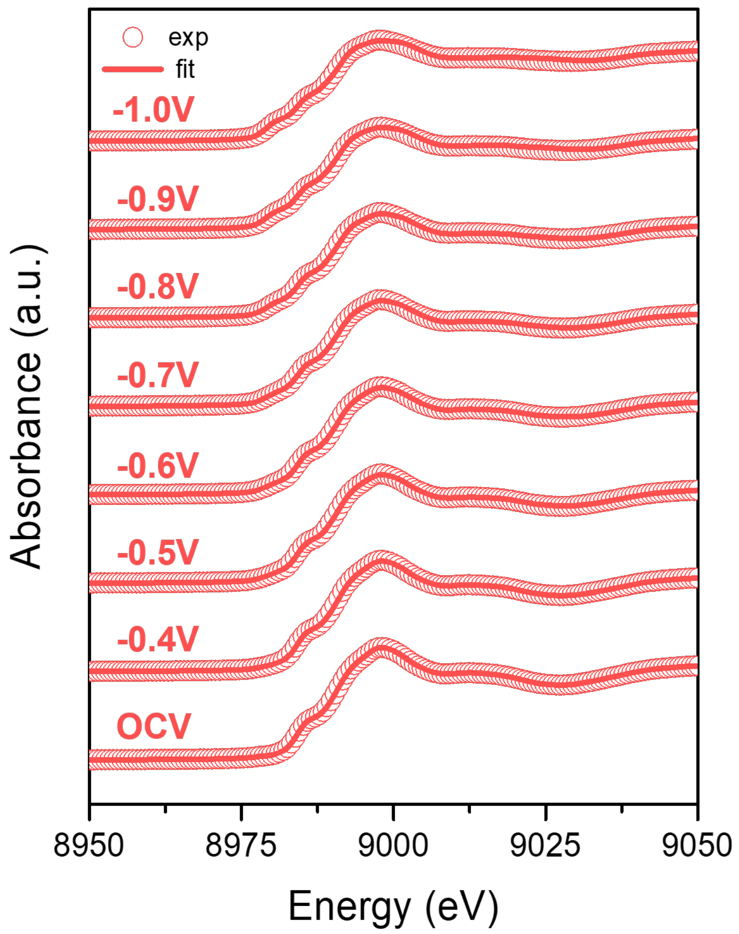

Supplementary Fig. 14. Linear combination fitting of the experimental curves for CuO. All potentials are reported versus RHE with 85% *iR*-correction (*i*, current; *R*, solution resistance). Source data are provided as a Source Data file.

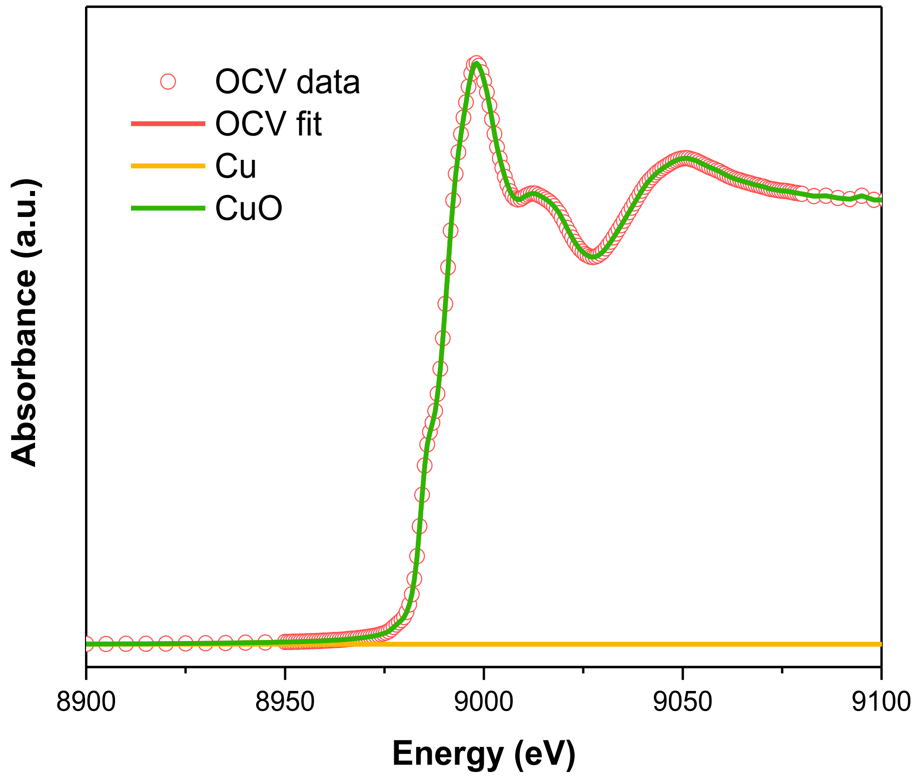

Supplementary Fig. 15. XANES fitting of CuO at OCV. Source data are provided as a Source Data file.

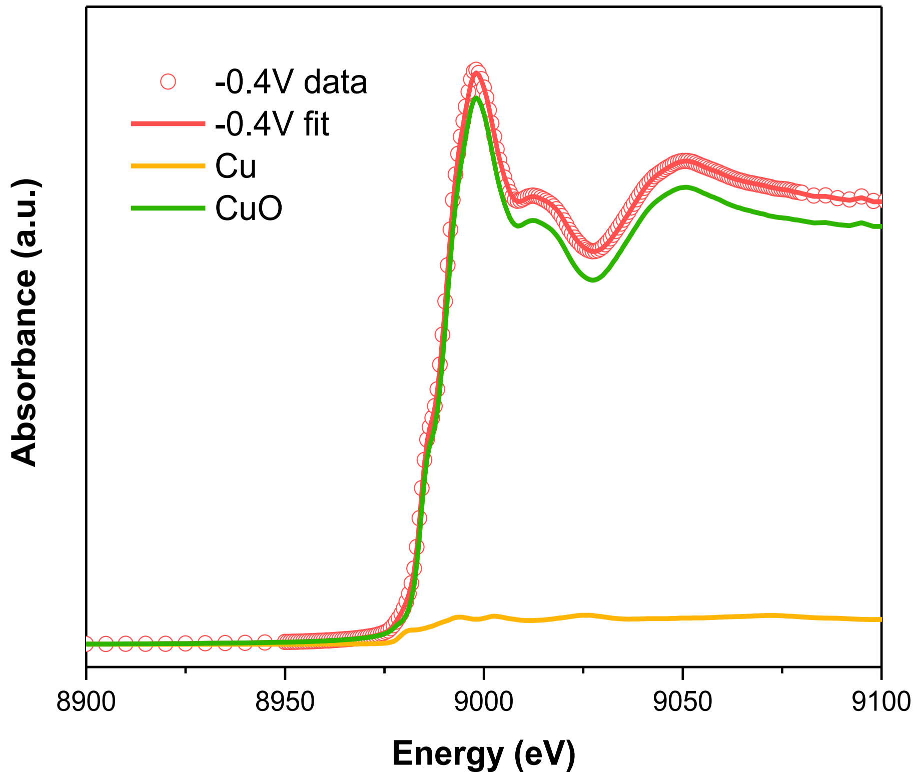

Supplementary Fig. 16. XANES fitting of CuO at −0.4V versus RHE with 85% *iR*-correction (*i*, current; *R*, solution resistance). Source data are provided as a Source Data file.

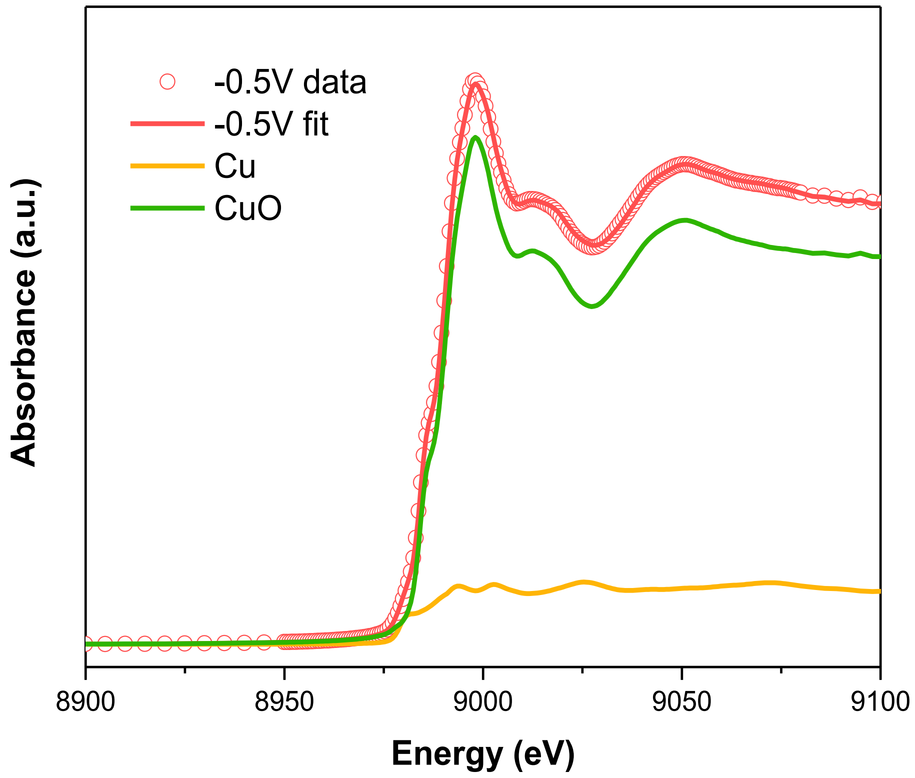

Supplementary Fig. 17. XANES fitting of CuO at −0.5V versus RHE with 85% *iR*-correction (*i*, current; *R*, solution resistance). Source data are provided as a Source Data file.

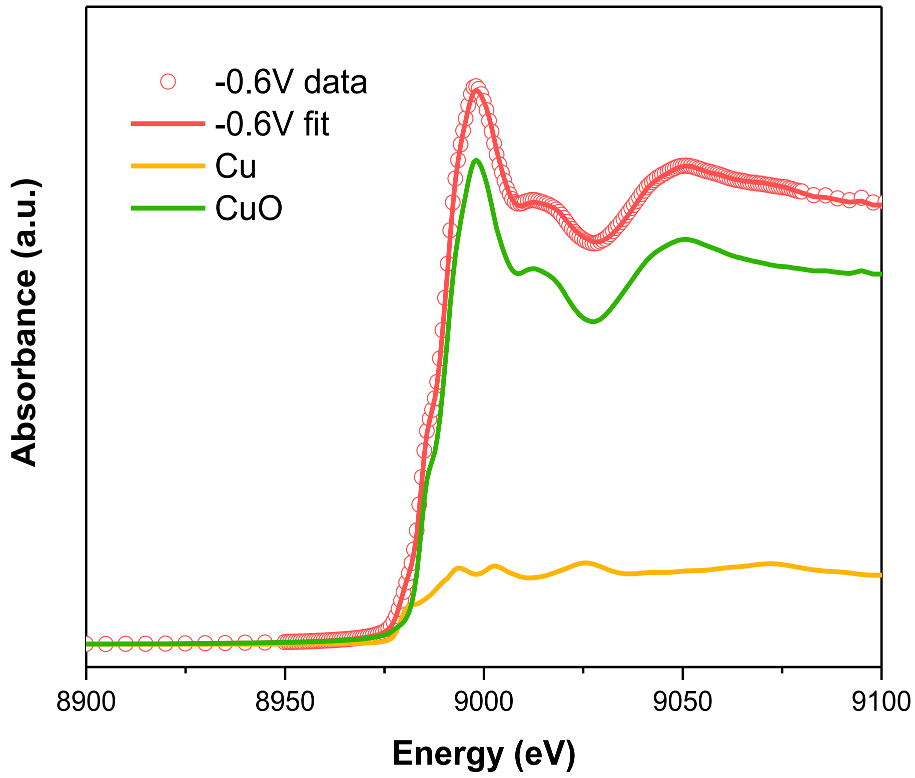

Supplementary Fig. 18. XANES fitting of CuO at −0.6V versus RHE with 85% *iR*-correction (*i*, current; *R*, solution resistance). Source data are provided as a Source Data file.

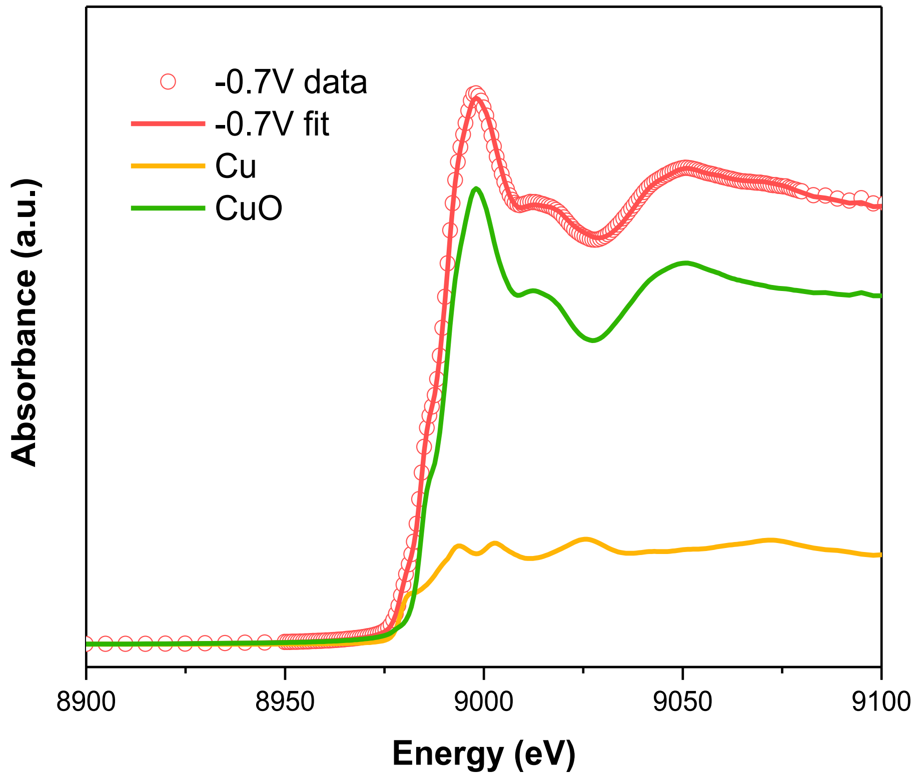

Supplementary Fig. 19. XANES fitting of CuO at −0.7V versus RHE with 85% *iR*-correction (*i*, current; *R*, solution resistance). Source data are provided as a Source Data file.

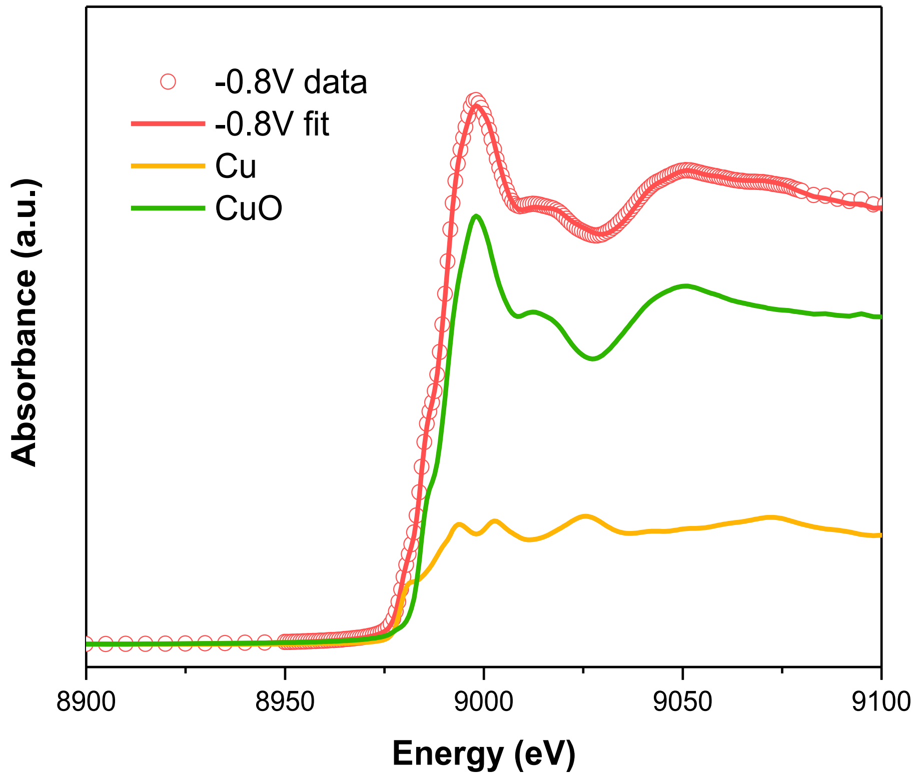

Supplementary Fig. 20. XANES fitting of CuO at −0.8V versus RHE with 85% *iR*-correction (*i*, current; *R*, solution resistance). Source data are provided as a Source Data file.

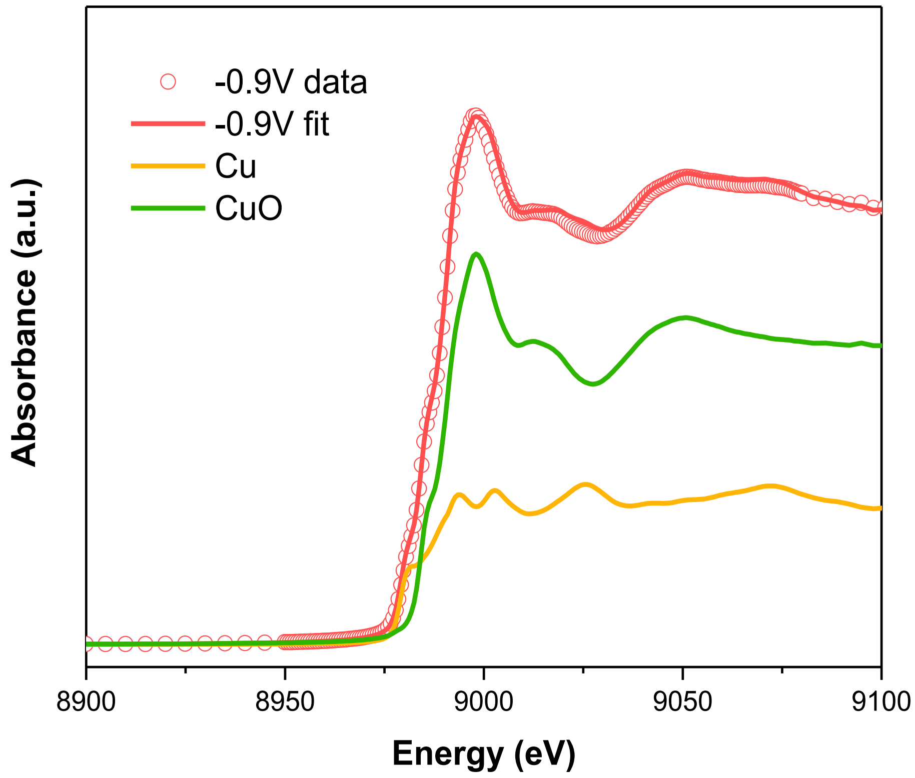

Supplementary Fig. 21. XANES fitting of CuO at −0.9V versus RHE with 85% *iR*-correction (*i*, current; *R*, solution resistance). Source data are provided as a Source Data file.

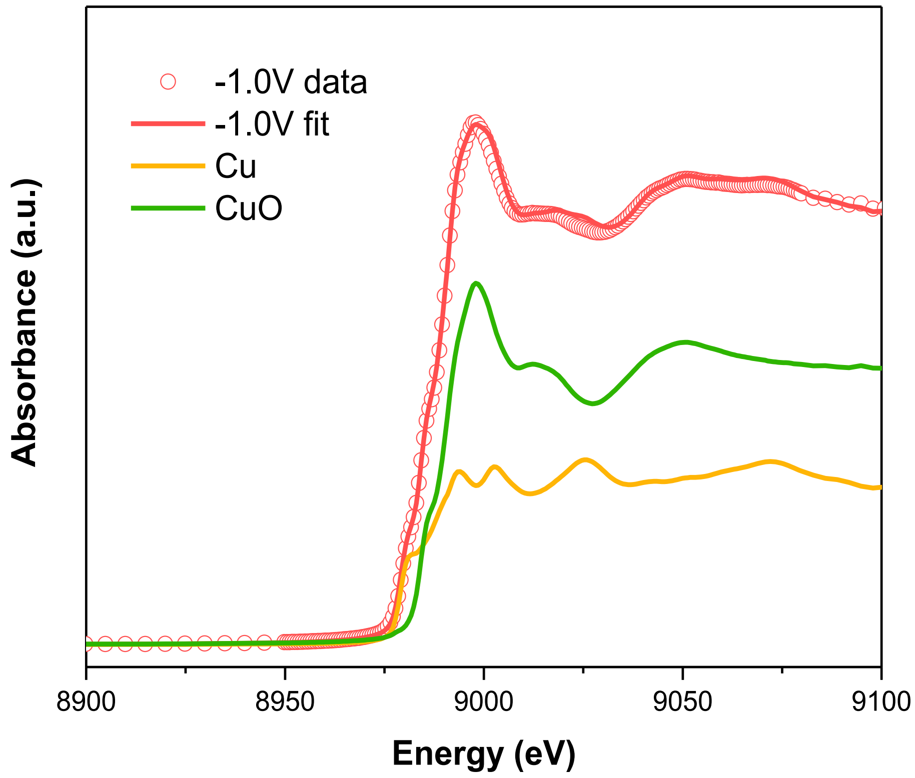

Supplementary Fig. 22. XANES fitting of CuO at −1.0V versus RHE with 85% *iR*-correction (*i*, current; *R*, solution resistance). Source data are provided as a Source Data file.

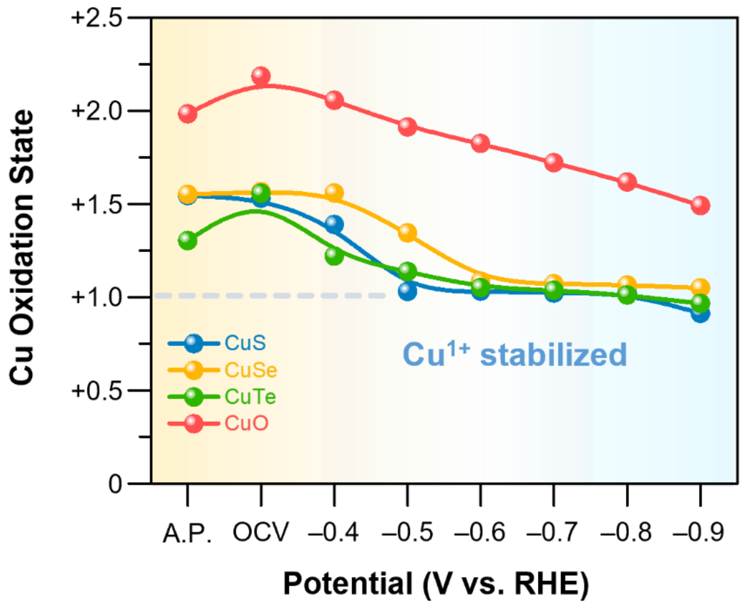

Supplementary Fig. 23. The Cu oxidation state as a function of potential for CuXs and CuO. All potentials are reported versus RHE with 85% *iR*-correction (*i*, current; *R*, solution resistance). Source data are provided as a Source Data file.

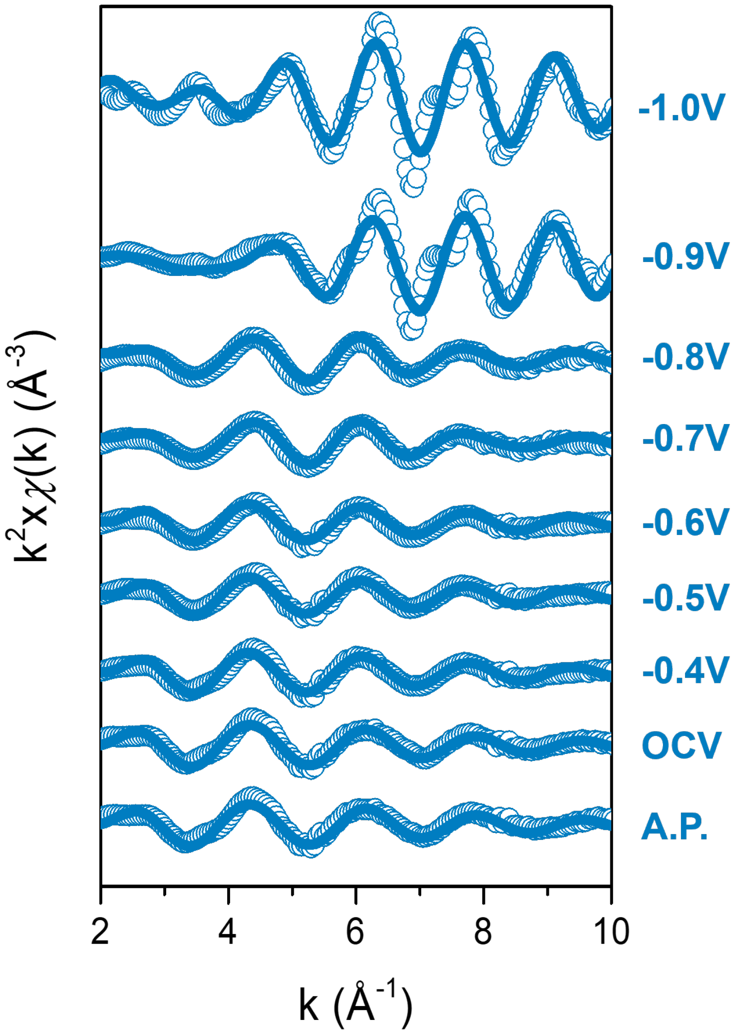

Supplementary Fig. 24. *In situ* k^2-weighted FT-EXAFS fitting curves in k-space of Cu K-edge for CuS. All potentials are reported versus RHE with 85% *iR*-correction (*i*, current; *R*, solution resistance). Source data are provided as a Source Data file.

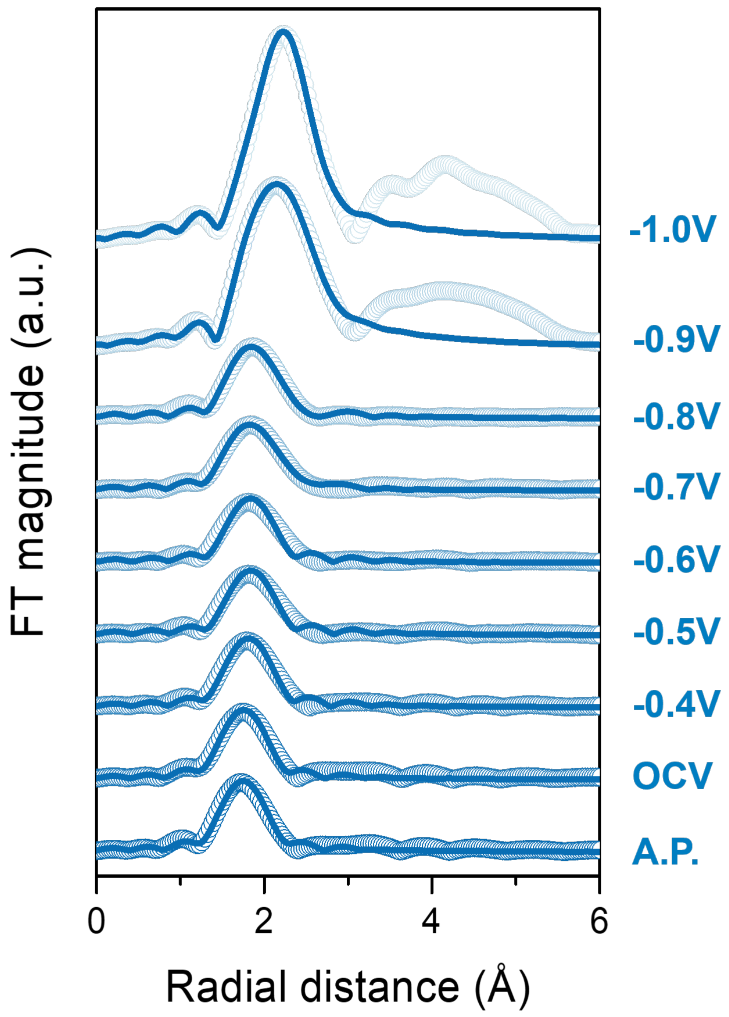

Supplementary Fig. 25. *In situ* k^2-weighted FT-EXAFS fitting curves in R-space of Cu K-edge for CuS. All potentials are reported versus RHE with 85% *iR*-correction (*i*, current; *R*, solution resistance). Source data are provided as a Source Data file.

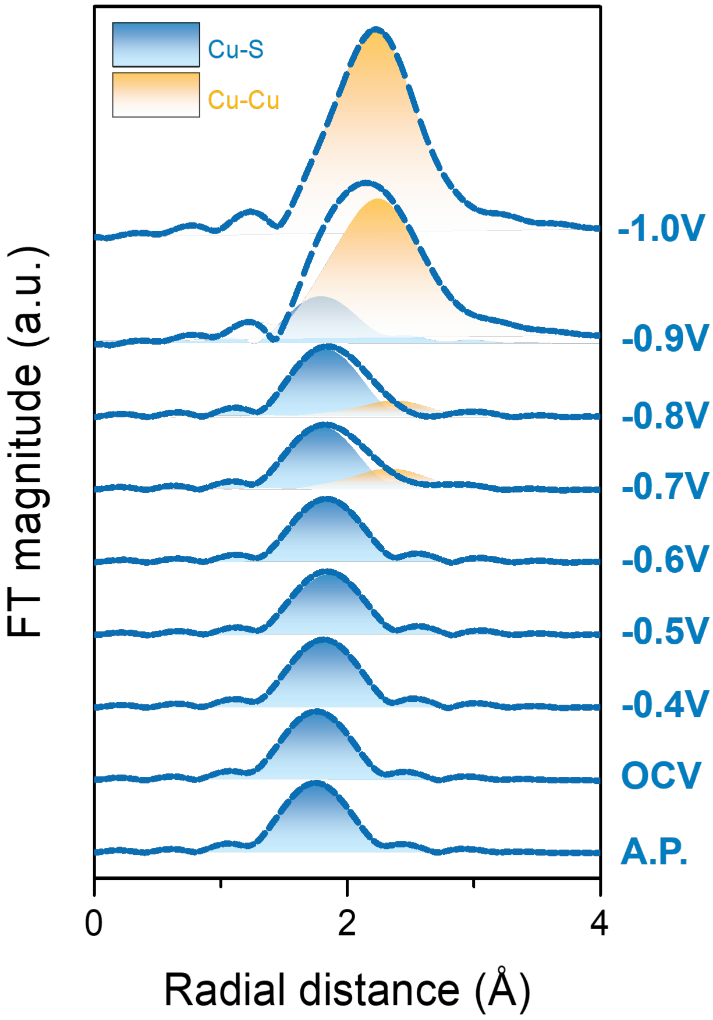

Supplementary Fig. 26. Deconvoluted k^2-weighted FT-EXAFS fitting of CuS. Peaks are assigned to Cu–S bond (blue) and Cu–Cu bond (yellow) from low to high radial distance. All potentials are reported versus RHE with 85% *iR*-correction (*i*, current; *R*, solution resistance). Source data are provided as a Source Data file.

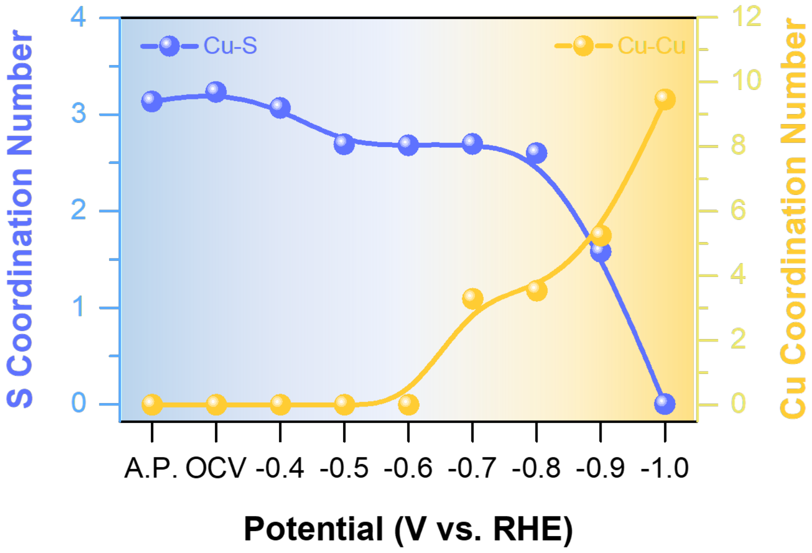

Supplementary Fig. 27. The S and Cu coordination number at Cu K-edge as a function of potential for CuS. All potentials are reported versus RHE with 85% *iR*-correction (*i*, current; *R*, solution resistance). Source data are provided as a Source Data file.

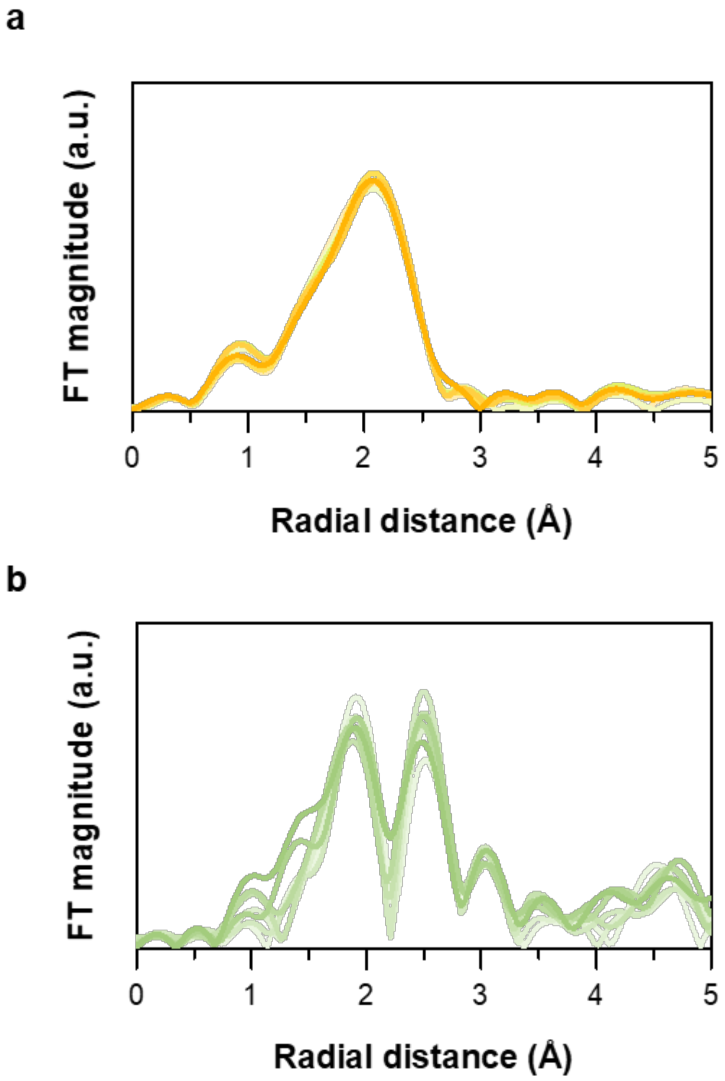

Supplementary Fig. 28. *In situ* k^2-weighted FT-EXAFS of Cu K-edge for **a,** CuSe, and **b,** CuTe. Source data are provided as a Source Data file.

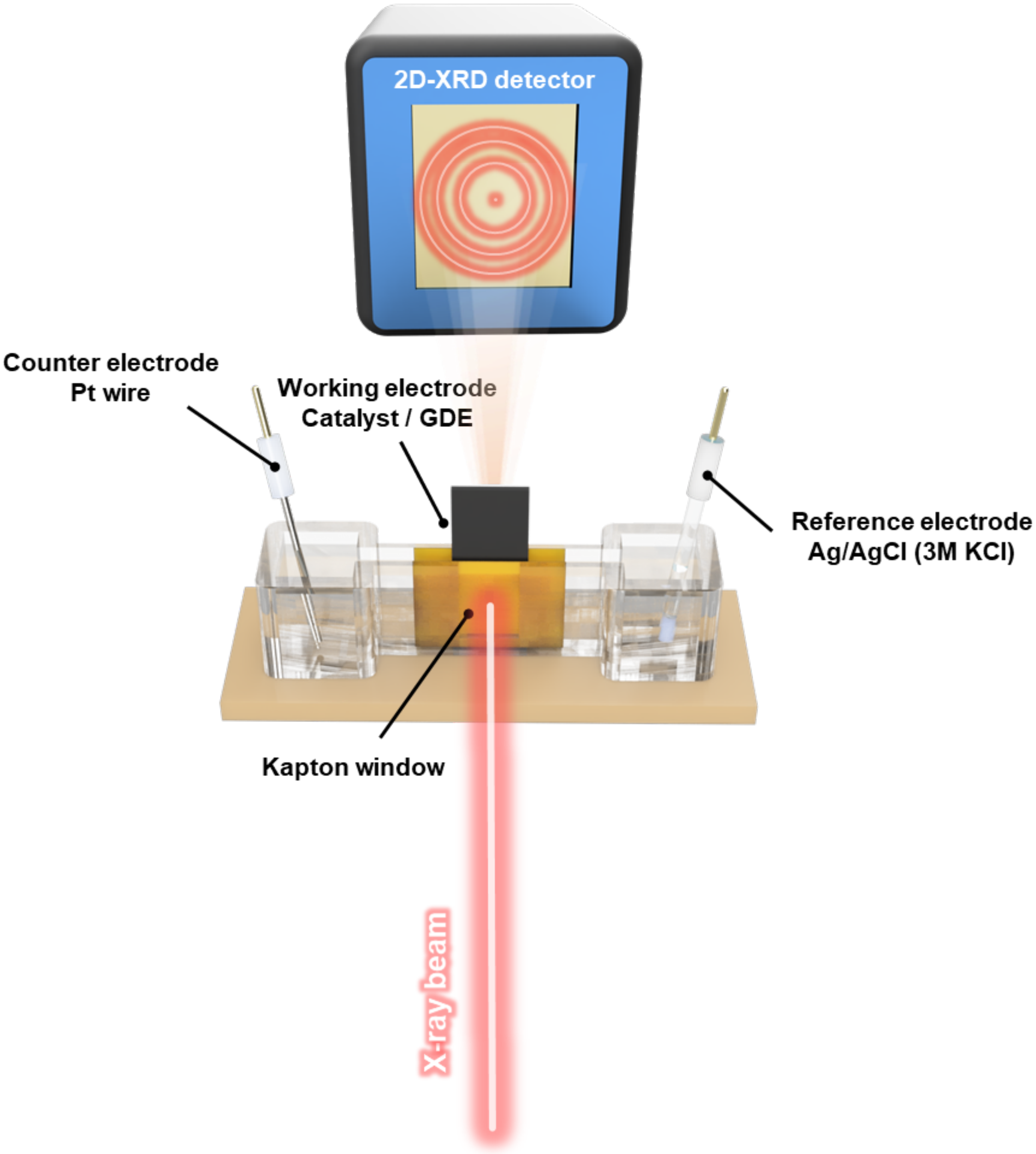

Supplementary Fig. 29. Experimental setup for *in situ* XRD measurements.

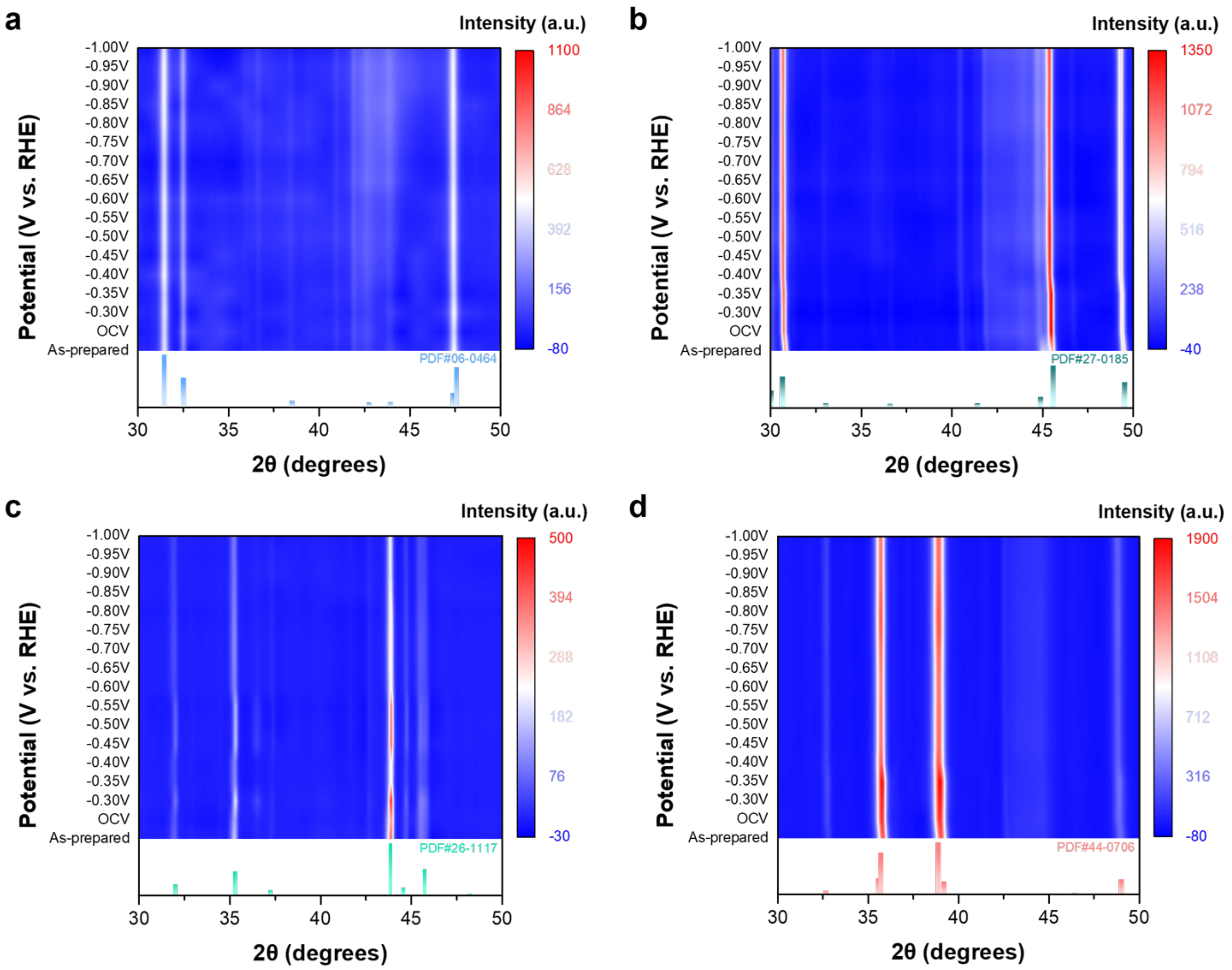

Supplementary Fig. 30. *In situ* XRD mapping as a function of potential of **a,** CuS **b,** CuSe **c,** CuTe, and **d,** CuO. All potentials are reported versus RHE with 85% *iR*-correction (*i*, current; *R*, solution resistance). Source data are provided as a Source Data file.

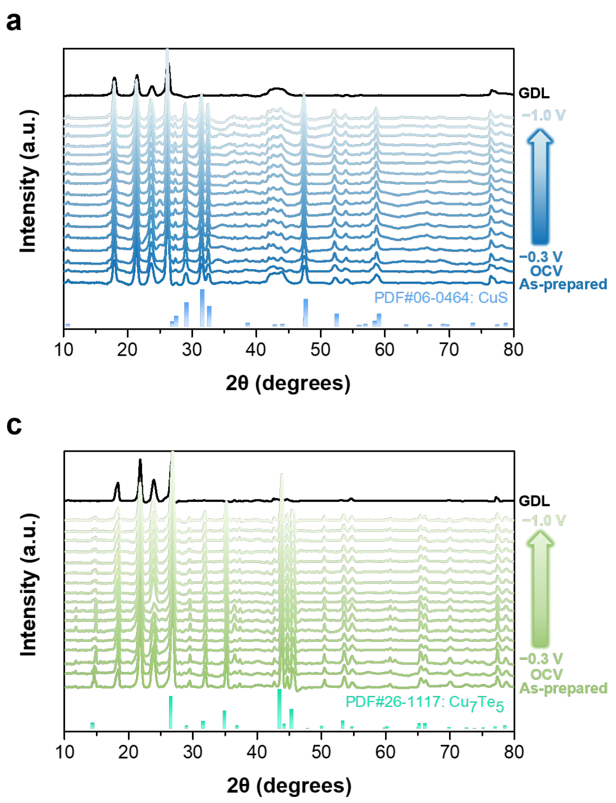

Supplementary Fig. 31. *In situ* XRD spectra of each sample: **a,** CuS, **b,** CuSe, **c,** CuTe, and **d,** CuO. All potentials are reported versus RHE with 85% *iR*-correction (*i*, current; *R*, solution resistance). Source data are provided as a Source Data file.

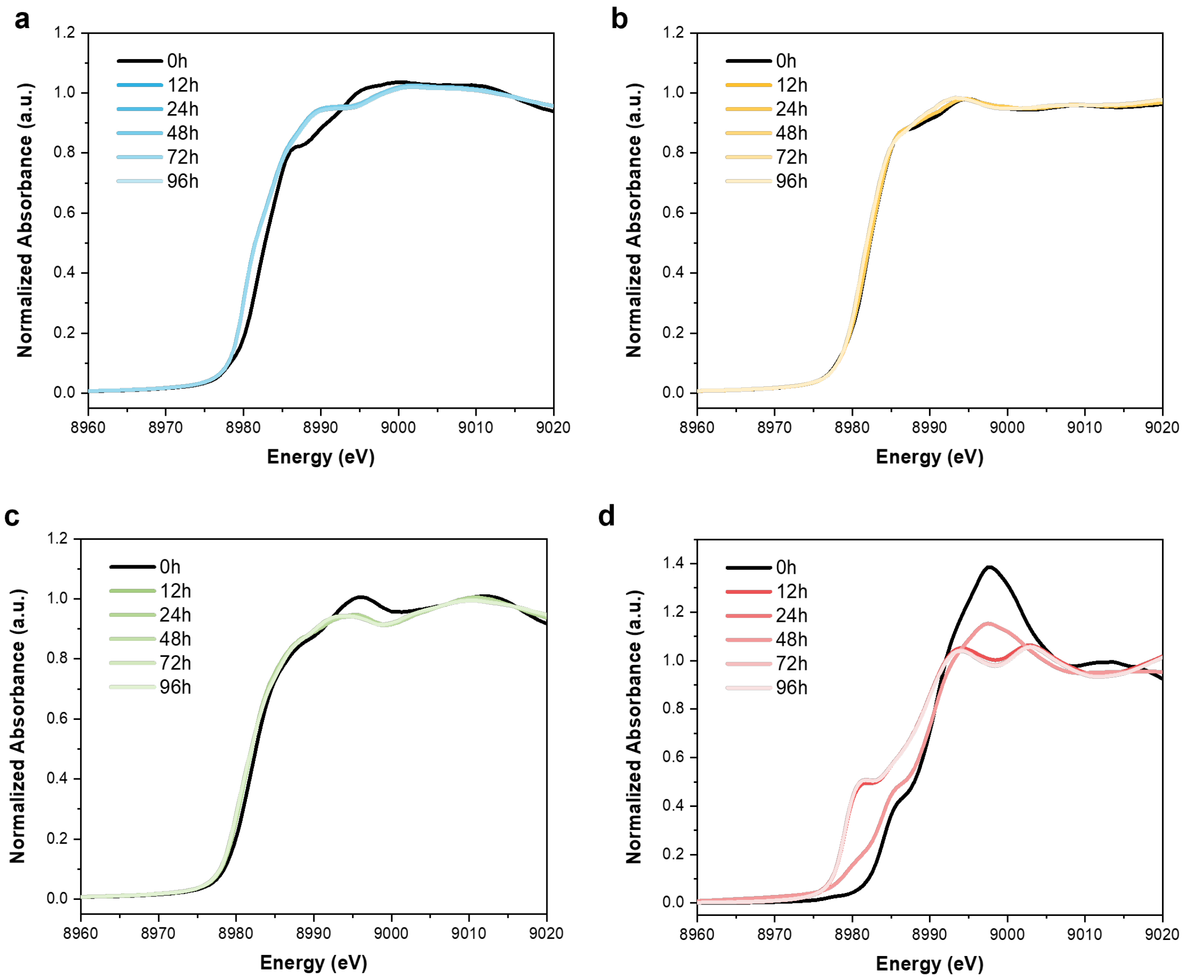

Supplementary Fig. 32. Real-time *in situ* XANES spectra at −0.6 V versus RHE with 85% *iR*-correction (*i*, current; *R*, solution resistance) of each sample: **a,** CuS, **b,** CuSe, **c,** CuTe, and **d,** CuO. Source data are provided as a Source Data file.

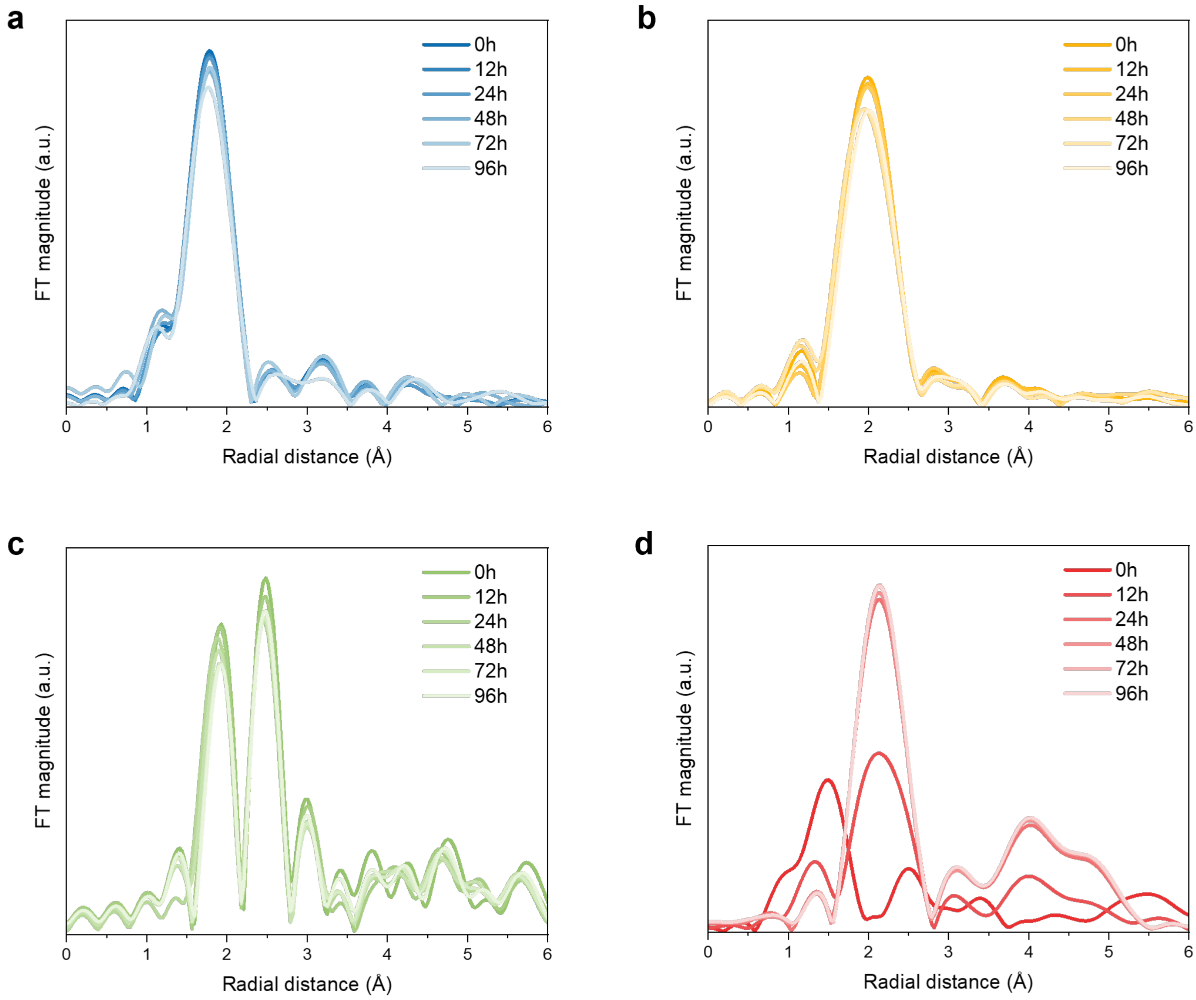

Supplementary Fig. 33. Real-time *in situ* EXAFS spectra at −0.6 V versus RHE with 85% *iR*-correction (*i*, current; *R*, solution resistance) of each sample: **a,** CuS, **b,** CuSe, **c,** CuTe, and **d,** CuO. Source data are provided as a Source Data file.

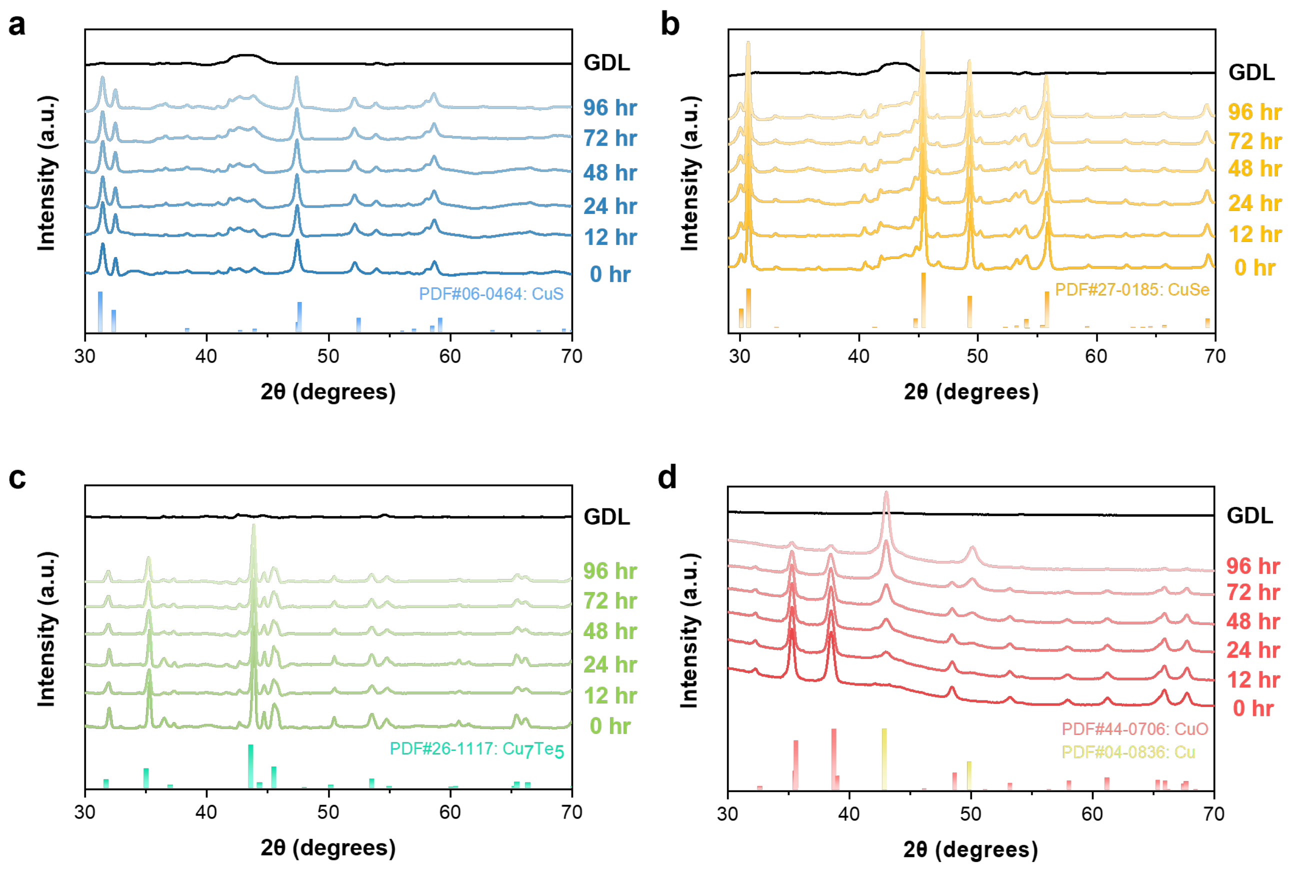

Supplementary Fig. 34. *Ex situ* XRD spectra at −0.6 V versus RHE with 85% *iR*-correction (*i*, current; *R*, solution resistance) of each sample: **a,** CuS, **b,** CuSe, **c,** CuTe, and **d,** CuO. Source data are provided as a Source Data file.

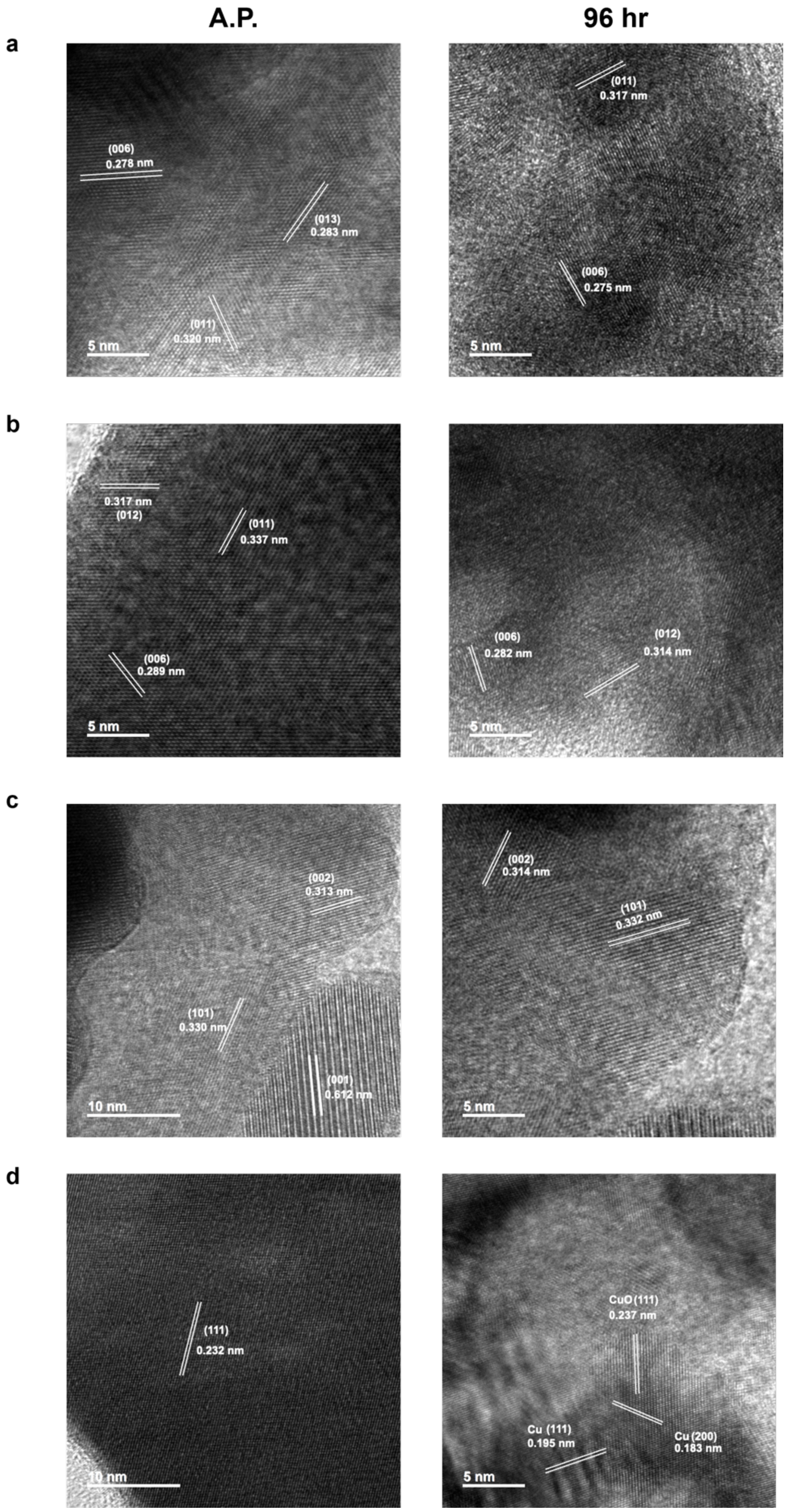

Supplementary Fig. 35. High-resolution transmission electron microscopy (HR-TEM) image of each sample: **a,** CuS, **b,** CuSe, **c,** CuTe, and **d,** CuO (left: pre-catalyst; right: post-catalyst at −0.6 V versus RHE with 85% *iR*-correction (*i*, current; *R*, solution resistance)).

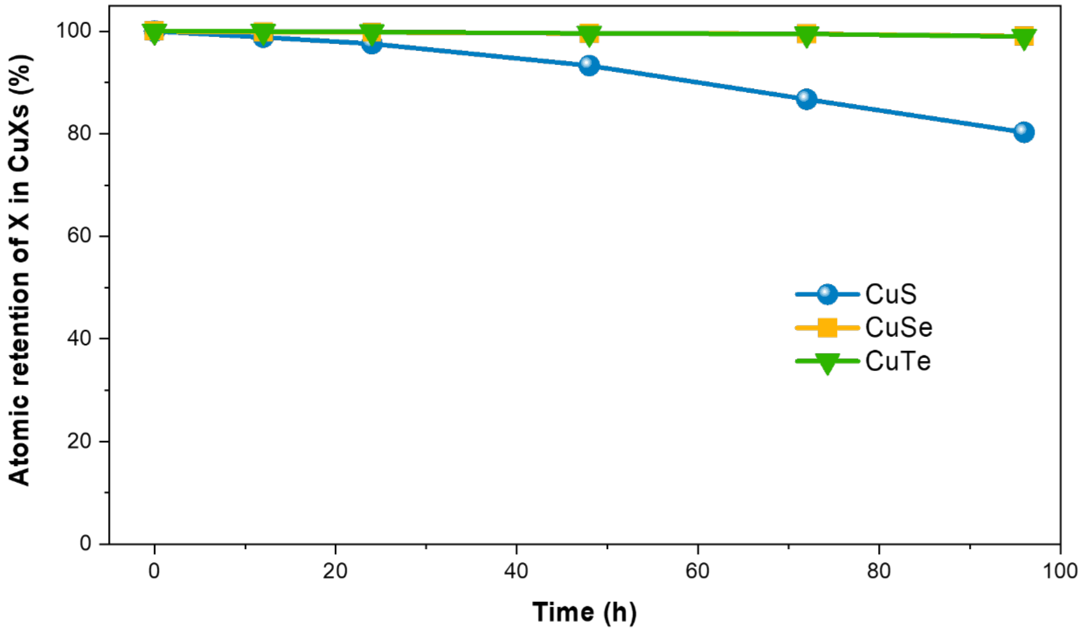

Supplementary Fig. 36. Inductively coupled plasma mass spectrometry (ICP-MS) analysis as a function of time for **a,** CuS, **b,** CuSe, **c,** CuTe, and **d,** CuO. Source data are provided as a Source Data file.

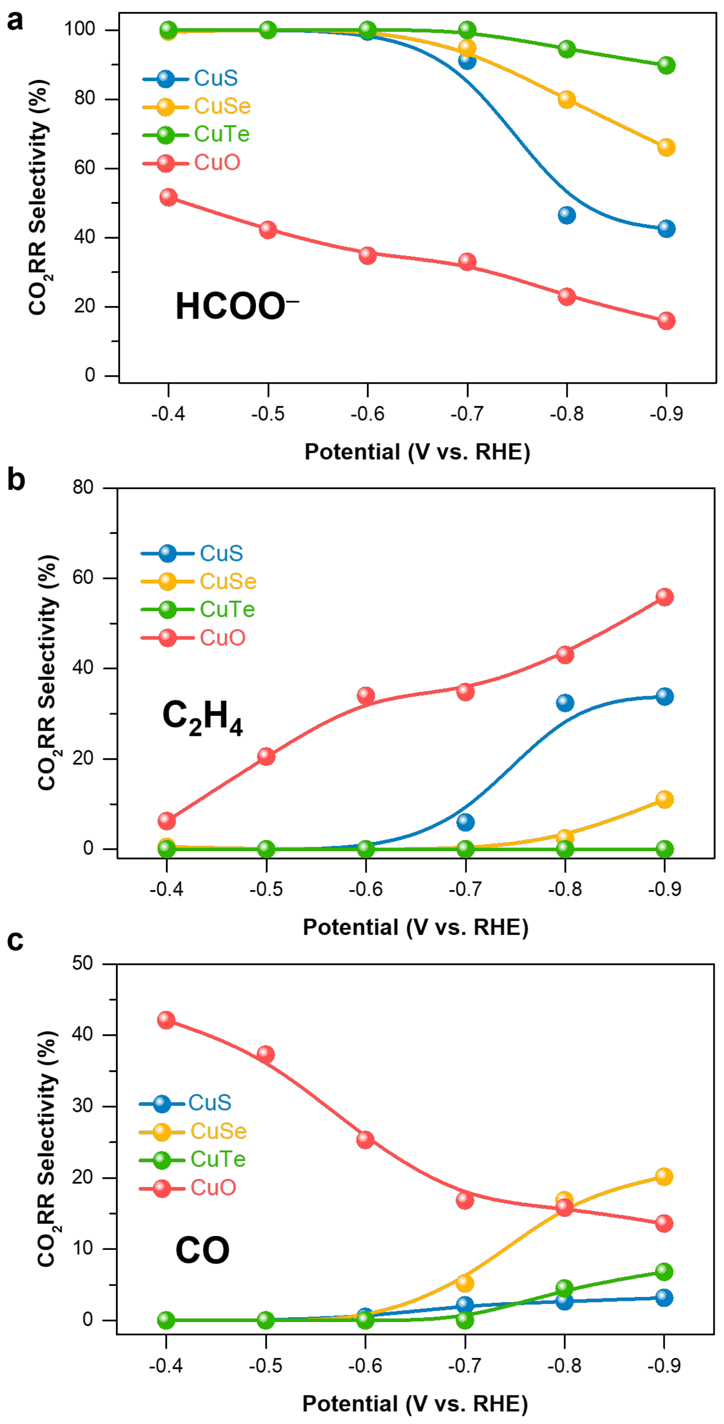

Supplementary Fig. 37. CO_2RR selectivity of **a,** formate, **b,** ethylene, and **c,** carbon monoxide. All potentials are reported versus RHE with 85% *iR*-correction (*i*, current; *R*, solution resistance). Source data are provided as a Source Data file.

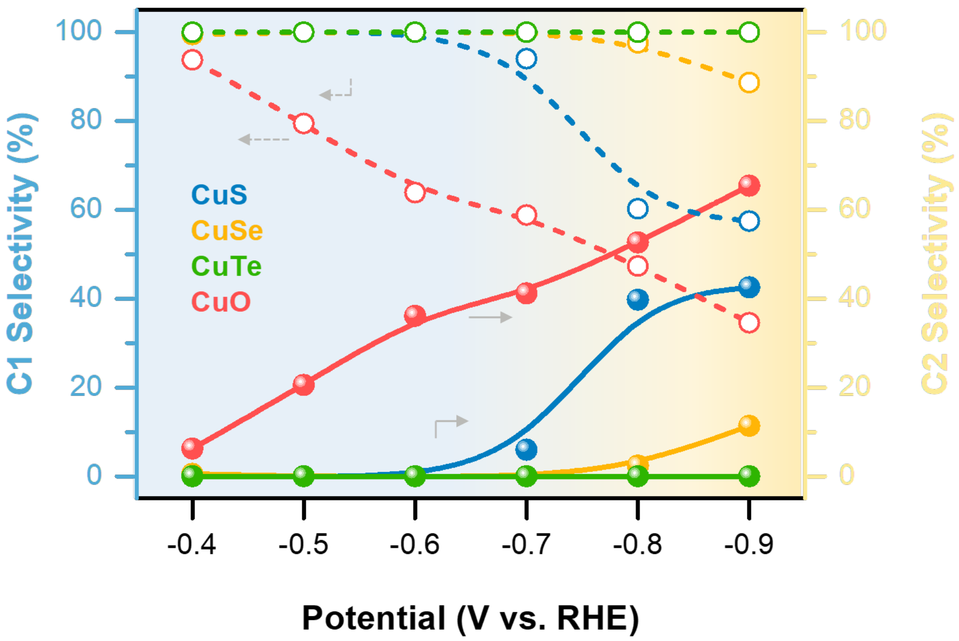

Supplementary Fig. 38. Selectivity of C1 (solid lines) and C2 (dash lines) products as a function of potential for CuXs and CuO. All potentials are reported versus RHE with 85% *iR*-correction (*i*, current; *R*, solution resistance). Source data are provided as a Source Data file.

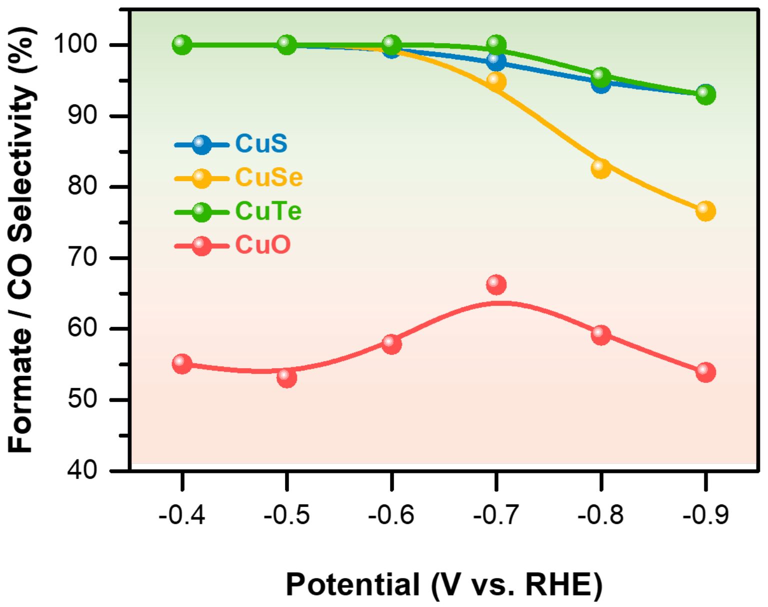

Supplementary Fig. 39. Selectivity of formate versus carbon monoxide as a function of potential for CuXs and CuO. All potentials are reported versus RHE with 85% *iR*-correction (*i*, current; *R*, solution resistance). Source data are provided as a Source Data file.

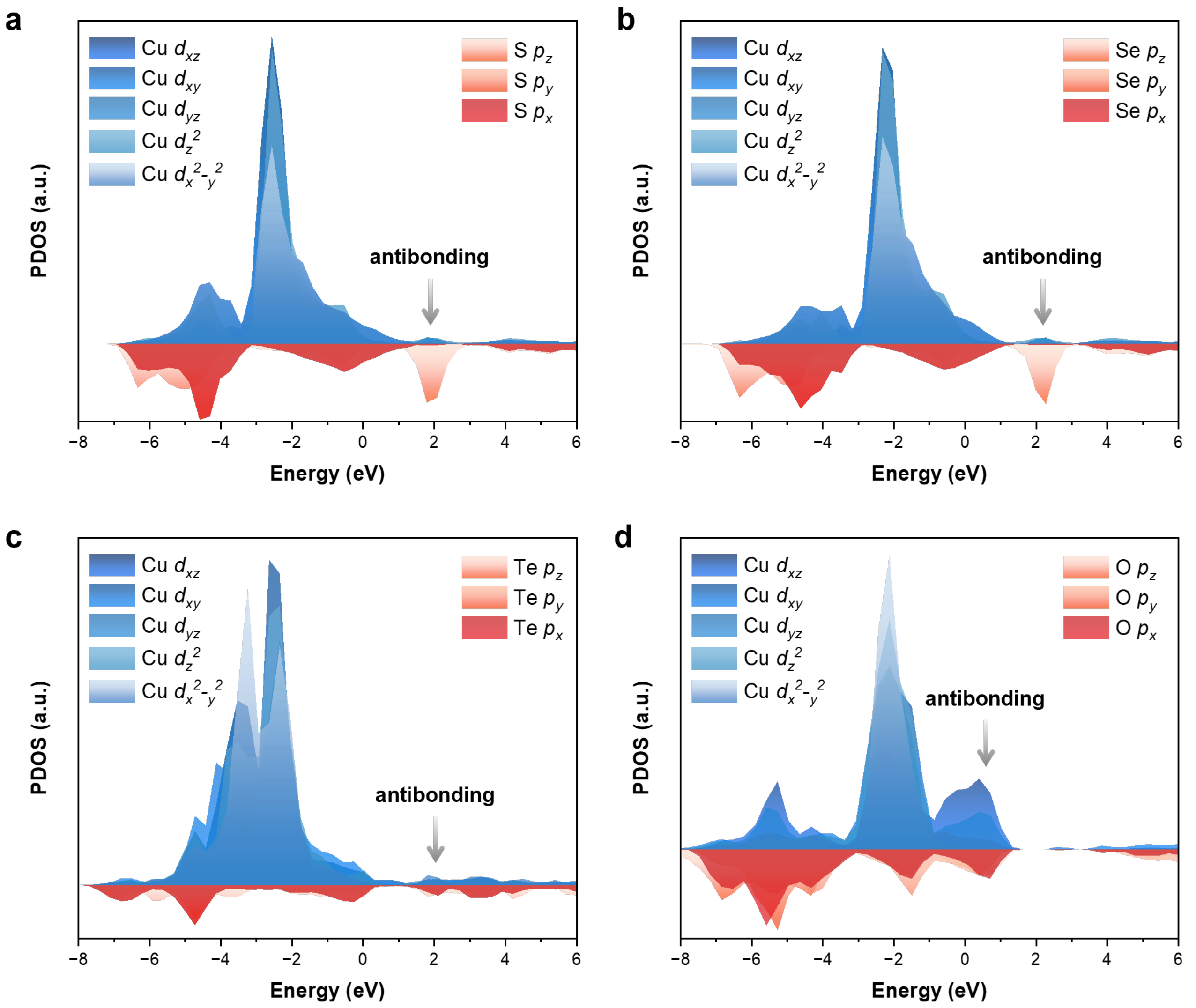

Supplementary Fig. 40. DFT calculations of projected density of states (PODS) analysis on Cu 3*d* orbitals for **a,** CuS, **b,** CuSe, **c,** CuTe, and **d,** CuO. Source data are provided as a Source Data file.

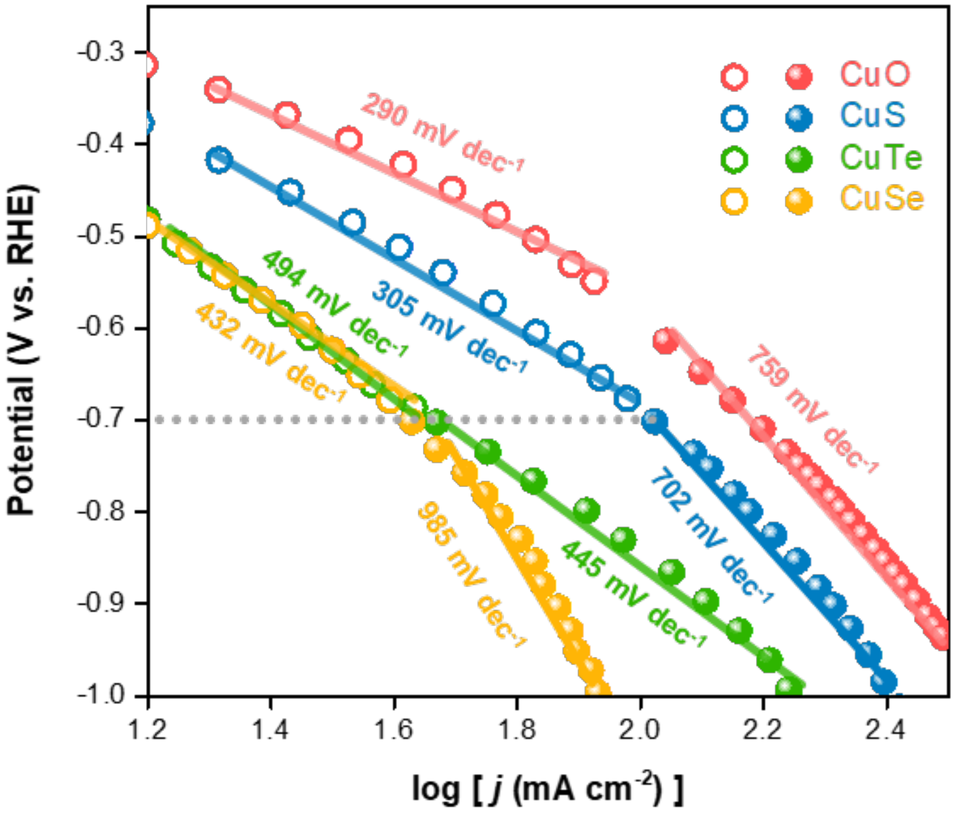

Supplementary Fig. 41. CO_2RR tafel plot for CuXs and CuO. Source data are provided as a Source Data file.

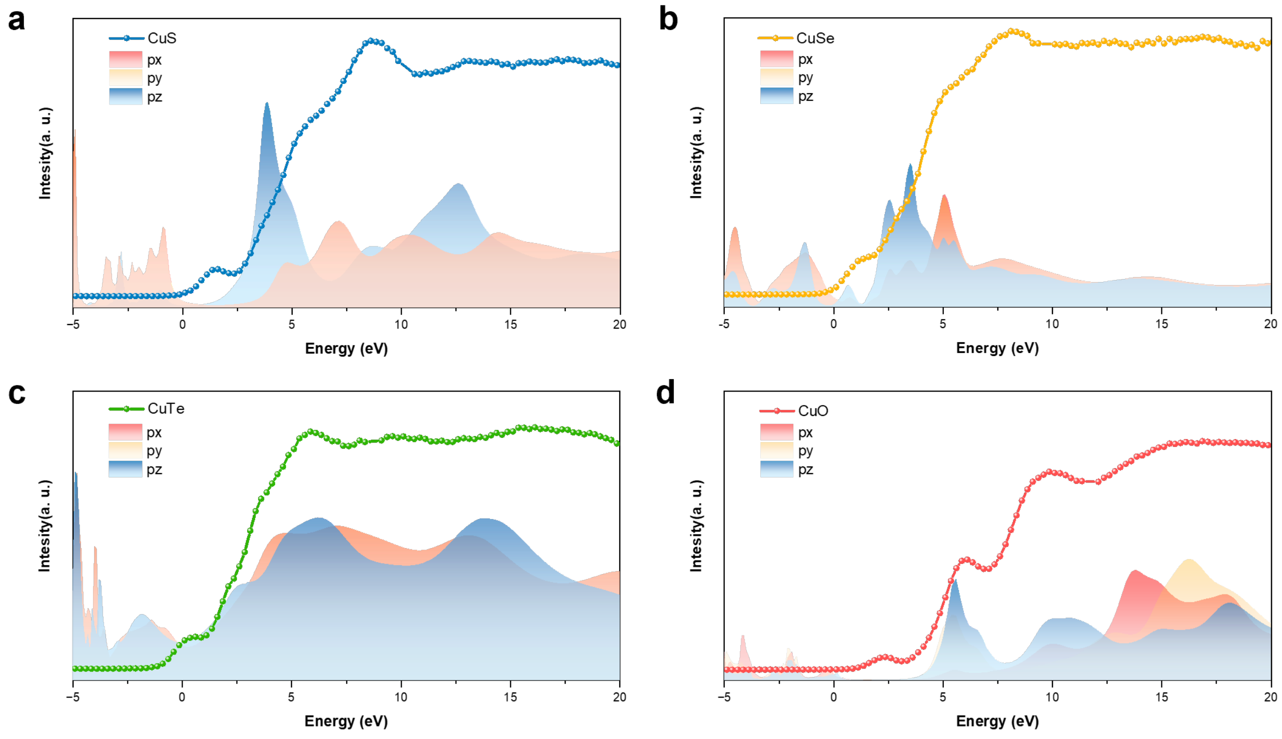

Supplementary Fig. 42. DFT calculations of projected density of states (PODS) analysis on Cu 4*p* orbitals overlaid with corresponding HERFD-XAS spectra for **a,** CuS, **b,** CuSe, **c,** CuTe, and **d,** CuO. Source data are provided as a Source Data file.

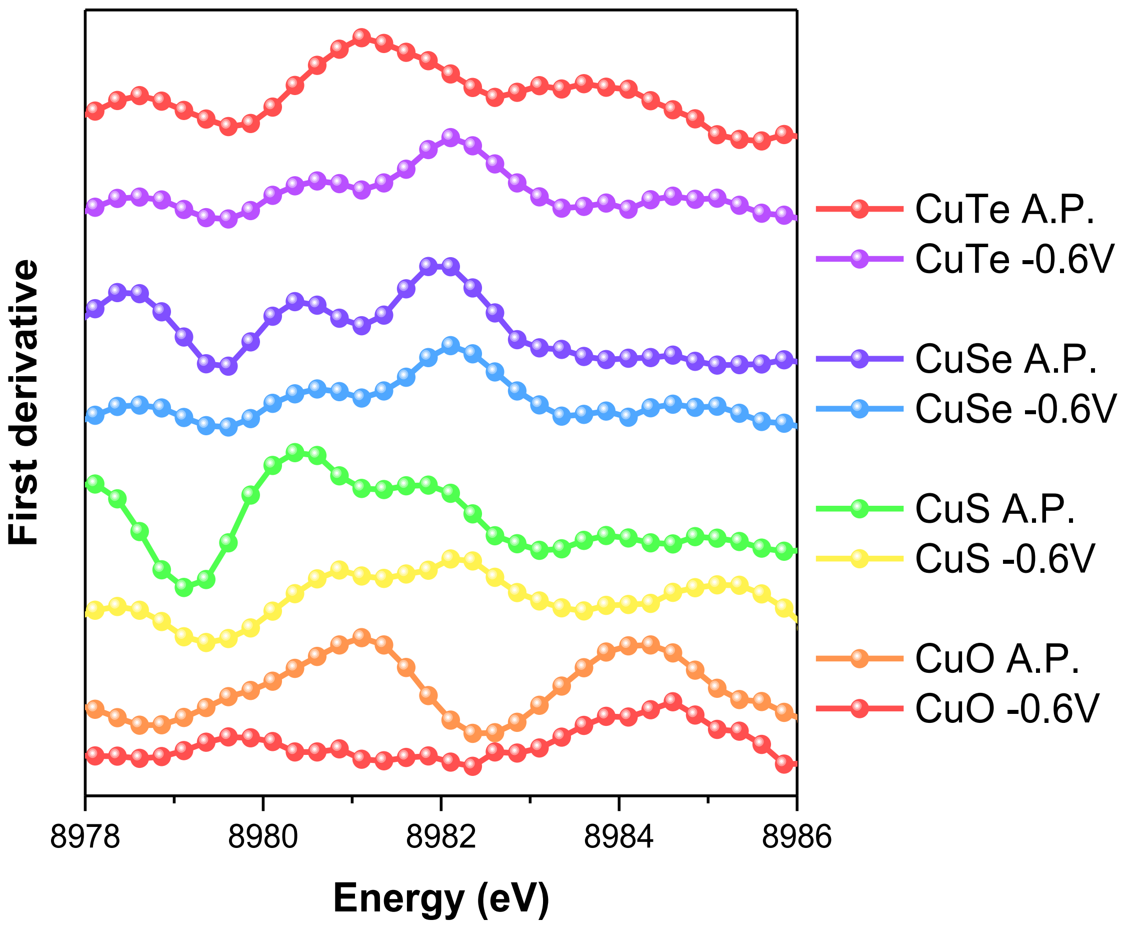

Supplementary Fig. 43. First derivative of *in situ* HERFD-XAS Cu K-edge spectra. All potentials are reported versus RHE with 85% *iR*-correction (*i*, current; *R*, solution resistance). Source data are provided as a Source Data file.

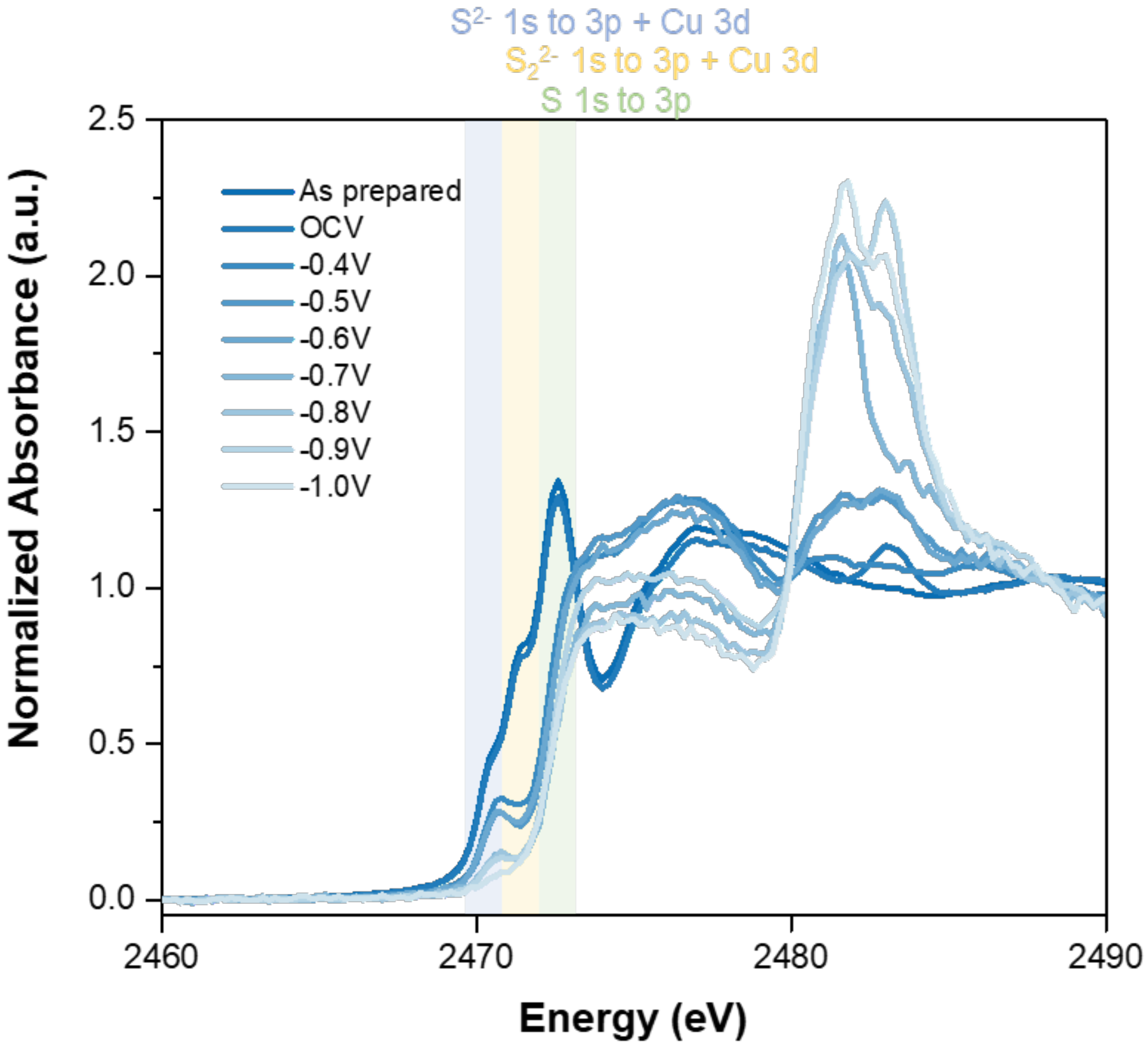

Supplementary Fig. 44. *Ex situ* XAS S K-edge spectra of CO_2RR for CuS. All potentials are reported versus RHE with 85% *iR*-correction (*i*, current; *R*, solution resistance). Source data are provided as a Source Data file.

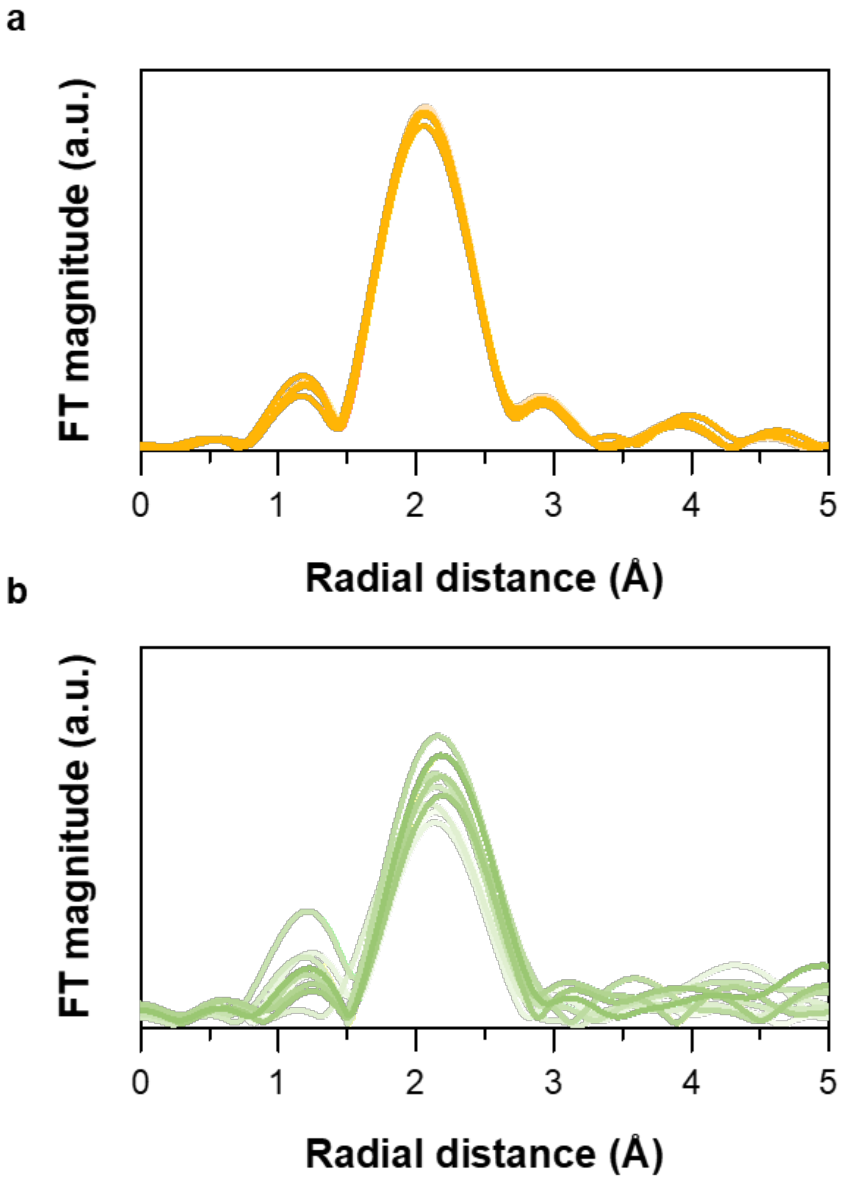

Supplementary Fig. 45. *In situ* k^2-weighted FT-EXAFS of **a,** Se K-edge for CuSe, and **b,** Te K-edge for CuTe. Source data are provided as a Source Data file.

Supplementary Tables

Supplementary Table 1. SEM-EDS element analysis of CuS, CuSe, CuTe, and CuO.

**Catalyst**	**Atomic ratio (%)**	
CuS	**Cu**	**S**
	50.9	49.1
CuSe	**Cu**	**Se**
	48.7	51.3
CuTe	**Cu**	**Te**
	64.9	35.1
CuO	**Cu**	**O**
	33.7	66.3

Supplementary Table 2. Cu K-edge position and relative oxidation state of CuS, CuSe, CuTe, and CuO.

Catalyst	Edge position	Relative O.S.
CuS	8981.91	+ 1.54
CuSe	8981.93	+ 1.55
CuTe	8981.07	+ 1.30
CuO	8983.44	+ 1.98

Supplementary Table 3. Fitting parameters of k^2-weighted FT-EXAFS at Cu K-edge for CuS, CuSe, CuTe, and CuO. The EXAFS oscillation was fitted by Artemis software

Catalyst	Path	CN [a]	R (Å) [b]	ΔE_0 (eV) [c]	$\Delta\sigma^2$ ($Å^2$) [d]	S_0^2 [e]	R-factor [f]
CuS	Cu–S	3.1±0.4	2.24±0.01	1.1±1.2	0.0078±0 .0021	0.8	0.009
CuSe	Cu–Se	3.4±0.9	2.37±0.02	3.9±3.2	0.0084±0.0022	0.85	0.021
CuTe	Cu–Te	2.2±0.7	2.58±0.06	–2.2±5.5	0.0088±0.0081	0.85	0.049
	Cu–Cu	1.9±0.8	2.58±0.06				
CuO	Cu–O	3.1±0.3	1.95±0.01	7.0±1.1	0.0029±0.0014	0.85	0.005

a, CN, coordination number; **b,** R, interatomic distance of the bond length between absorber and backscatter atoms; **c,** ΔE_0, edge-energy shift; **d,** $\Delta\sigma^2$, Debye-Waller factor accounts for both thermal and structural disorders in the absorber-scatter distance; **e,** S_0^2, passive electron reduction factor; **f,** R-factor indicates the quality of EXAFS fitting.

Supplementary Table 4. Composition profile of linear combination fitting of CuO.

Condition	Cu^0 (%)	Cu^{1+} (%)	Cu^{2+} (%)
OCV	0	0	100.00
–0.4V	5.89	0	94.11
–0.5V	12.47	0	87.53
–0.6V	16.47	0	83.53
–0.7V	21.28	0	78.72
–0.8V	25.97	0	74.03
–0.9V	31.72	0	68.28
–1.0V	36.81	0	63.19

Supplementary Table 5. Linear combination fitting parameters of CuO.

Condition	Cu^0	Cu^{1+}	Cu^{2+}	R-factor (10^{-4})
OCV	0.000±0.000	0	1.000±0.008	0.01
−0.4V	0.059±0.003	0	0.941±0.009	1.10
−0.5V	0.125±0.003	0	0.875±0.009	0.98
−0.6V	0.165±0.003	0	0.835±0.009	1.12
−0.7V	0.213±0.004	0	0.787±0.009	1.76
−0.8V	0.260±0.004	0	0.740±0.009	1.90
−0.9V	0.317±0.004	0	0.683±0.009	2.29
−1.0V	0.368±0.004	0	0.632±0.009	1.90

Supplementary Table 6. Fitting parameters of *in situ* k^2-weighted FT-EXAFS at Cu K-edge for CuS. The EXAFS oscillation was fitted by Artemis software.

CuS	Path	CN [a]	R (Å) [b]	ΔE_0 (eV) [c]	$\Delta\sigma^2$ ($Å^2$) [d]	S_0^2 [e]	R-factor [f]
A.P.	Cu–S	3.1±0.4	2.24±0.01	1.1±1.2	0.0078±0 .0021	0.8	0.009
OCV	Cu–S	3.2±0.5	2.25±0.01	1.7±1.3	0.0084±0.0022	0.8	0.010
−0.4V	Cu–S	3.1±0.4	2.28±0.01	4.5±1.1	0.0077±0.0018	0.8	0.006
−0.5V	Cu–S	2.7±0.4	2.30±0.02	5.8±1.5	0.0069±0.0024	0.8	0.011
−0.6V	Cu–S	2.7±0.5	2.30±0.02	5.6±1.7	0.0068±0.0026	0.8	0.013
−0.7V	Cu–S	2.7±0.5	2.27±0.02	3.8±1.6	0.0055±0.0029	0.8	0.008
	Cu–Cu	3.3±2.2	2.67±0.02				
−0.8V	Cu–S	2.6±0.5	2.29±0.02	5.4±1.6	0.0051±0.0026	0.8	0.010
	Cu–Cu	3.5±2.5	2.68±0.02				
−0.9V	Cu–S	1.6±0.3	2.25±0.02	4.8±1.4	0.0059±0.0022	0.8	0.003
	Cu–Cu	5.2±1.1	2.55±0.01				

a, CN, coordination number; **b,** R, interatomic distance of the bond length between absorber and backscatter atoms; **c,** ΔE_0, edge-energy shift; **d,** $\Delta\sigma^2$, Debye-Waller factor accounts for both thermal and structural disorders in the absorber-scatter distance; **e,** S_0^2, passive electron reduction factor; **f,** R-factor indicates the quality of EXAFS fitting.
