# Supplementary material for: Charge redistribution dynamics in chalcogenide-stabilized cuprous electrocatalysts unleash ampere-scale partial current toward formate production
Source: Nat Commun. 2025 Oct 24;16:9426. doi: 10.1038/s41467-025-64472-1 (PMC12552683; doi:10.1038/s41467-025-64472-1)
Supplement: Supplementary file 2 — Description of Additional Supplementary Files [file 41467_2025_64472_MOESM2_ESM.pdf]

Description of Additional Supplementary Files

File name: Supplementary Data 1

Description: Atomic positions of DFT models for copper chalcogenides.
