# Supplementary material for: Charge redistribution dynamics in chalcogenide-stabilized cuprous electrocatalysts unleash ampere-scale partial current toward formate production
Source: Nat Commun. 2025 Oct 24;16:9426. doi: 10.1038/s41467-025-64472-1 (PMC12552683; doi:10.1038/s41467-025-64472-1)
Supplement: Supplementary file 3 — Supplementary Data 1 [file 41467_2025_64472_MOESM3_ESM.zip › Supplementary Data 1.docx]

**Atomic positions of DFT models for copper chalcogenides**

**Coordinates of the Optimized Structures**

**Title (*OCHO/CuS)**

**1.00000000000000**

**7.5138239999999996 0.0000000000000000 0.0000000000000000**

**-3.7569119999999998 6.5071624600000000 0.0000000000000000**

**0.0000000000000000 0.0000000000000000 32.0000000000000000**

**S Cu C O H**

**24 24 1 2 1**

**Selective dynamics**

**Direct**

**0.1666666664105421 0.3333333328210770 0.18001539781249683# S**

**0.0000000000000000 0.0000000000000000 0.07423327875000043# S**

**0.3333333335894579 0.1666666671789230 0.43833812718749953# S**

**0.0000000000000000 0.0000000000000000 0.34208049812500013# S**

**0.0000000000000000 0.0000000000000000 0.27627192468749943# S**

**0.0000000000000000 0.0000000000000000 0.54411914437500293# S**

**0.6666666664105421 0.3333333328210770 0.18001539781249683# S**

**0.5000000000000000 0.0000000000000000 0.07423327875000043# S**

**0.8333333335894650 0.1666666671789230 0.43833812718749953# S**

**0.5000000000000000 0.0000000000000000 0.34208049812500013# S**

**0.5000000000000000 0.0000000000000000 0.27627192468749943# S**

**0.5000000000000000 0.0000000000000000 0.54411914437500293# S**

**0.1666666671789230 0.8333333343578460 0.18001539781249683# S**

**0.0000000000000000 0.5000000000000000 0.07423327875000043# S**

**0.3333333335894579 0.6666666671789230 0.43833812718749953# S**

**0.0000000000000000 0.5000000000000000 0.34208049812500013# S**

**0.0000000000000000 0.5000000000000000 0.27627192468749943# S**

**0.0000000000000000 0.5000000000000000 0.54411914437500293# S**

**0.6666666671789230 0.8333333343578460 0.18001539781249683# S**

**0.5000000000000000 0.5000000000000000 0.07423327875000043# S**

**0.8333333335894579 0.6666666671789230 0.43833812718749953# S**

**0.5000000000000000 0.5000000000000000 0.34208049812500013# S**

**0.5000000000000000 0.5000000000000000 0.27627192468749943# S**

**0.5000000000000000 0.5000000000000000 0.54411914437500293# S**

**0.1666666664105421 0.3333333328210770 0.45055698031249853# Cu**

**0.1666666664105421 0.3333333328210770 0.07905558218750033# Cu**

**0.3333333335894579 0.1666666671789230 0.16779544281249773# Cu**

**0.3333333335894579 0.1666666671789230 0.36732963531250113# Cu**

**0.1666666664105421 0.3333333328210770 0.25096364750000083# Cu**

**0.3333333335894579 0.1666666671789230 0.53929684093750303# Cu**

**0.6666666664105421 0.3333333328210770 0.45055698031249853# Cu**

**0.6666666664105421 0.3333333328210770 0.07905558218750033# Cu**

**0.8333333335894650 0.1666666671789230 0.16779544281249773# Cu**

**0.8333333335894650 0.1666666671789230 0.36732963531250113# Cu**

**0.6666666664105421 0.3333333328210770 0.25096364750000083# Cu**

**0.8333333335894650 0.1666666671789230 0.53929684093750303# Cu**

**0.1666666671789230 0.8333333343578460 0.45055698031249853# Cu**

**0.1666666671789230 0.8333333343578460 0.07905558218750033# Cu**

**0.3333333335894579 0.6666666671789230 0.16779544281249773# Cu**

**0.3333333335894579 0.6666666671789230 0.36732963531250113# Cu**

**0.1666666671789230 0.8333333343578460 0.25096364750000083# Cu**

**0.3333333335894579 0.6666666671789230 0.53929684093750303# Cu**

**0.6666666671789230 0.8333333343578460 0.45055698031249853# Cu**

**0.6666666671789230 0.8333333343578460 0.07905558218750033# Cu**

**0.8333333335894579 0.6666666671789230 0.16779544281249773# Cu**

**0.8333333335894579 0.6666666671789230 0.36732963531250113# Cu**

**0.6666666671789230 0.8333333343578460 0.25096364750000083# Cu**

**0.8333333335894579 0.6666666671789230 0.53929684093750303# Cu**

**0.4701253567389116 0.5829338031786122 0.62675738551916283# C**

**0.3715474907277283 0.6675733390180909 0.61947729199160763# O**

**0.5361461413829716 0.4912175949779183 0.59960190141615753# O**

**0.5224699190109874 0.5693213752230051 0.65838325792476843# H**

**Title (*COOH/CuS)**

**1.00000000000000**

**7.5138239999999996 0.0000000000000000 0.0000000000000000**

**-3.7569119999999998 6.5071624600000000 0.0000000000000000**

**0.0000000000000000 0.0000000000000000 32.0000000000000000**

**S Cu C O H**

**24 24 1 2 1**

**Selective dynamics**

**Direct**

**0.1666666664105421 0.3333333328210770 0.1800153978124968 # S**

**0.0000000000000000 0.0000000000000000 0.0742332787500004 # S**

**0.3333333335894579 0.1666666671789230 0.4383381271874995 # S**

**0.0000000000000000 0.0000000000000000 0.3420804981250001 # S**

**0.0000000000000000 0.0000000000000000 0.2762719246874994 # S**

**0.0000000000000000 0.0000000000000000 0.5441191443750029 # S**

**0.6666666664105421 0.3333333328210770 0.1800153978124968 # S**

**0.5000000000000000 0.0000000000000000 0.0742332787500004 # S**

**0.8333333335894650 0.1666666671789230 0.4383381271874995 # S**

**0.5000000000000000 0.0000000000000000 0.3420804981250001 # S**

**0.5000000000000000 0.0000000000000000 0.2762719246874994 # S**

**0.5000000000000000 0.0000000000000000 0.5441191443750029 # S**

**0.1666666671789230 0.8333333343578460 0.1800153978124968 # S**

**0.0000000000000000 0.5000000000000000 0.0742332787500004 # S**

**0.3333333335894579 0.6666666671789230 0.4383381271874995 # S**

**0.0000000000000000 0.5000000000000000 0.3420804981250001 # S**

**0.0000000000000000 0.5000000000000000 0.2762719246874994 # S**

**0.0000000000000000 0.5000000000000000 0.5441191443750029 # S**

**0.6666666671789230 0.8333333343578460 0.1800153978124968 # S**

**0.5000000000000000 0.5000000000000000 0.0742332787500004 # S**

**0.8333333335894579 0.6666666671789230 0.4383381271874995 # S**

**0.5000000000000000 0.5000000000000000 0.3420804981250001 # S**

**0.5000000000000000 0.5000000000000000 0.2762719246874994 # S**

**0.5000000000000000 0.5000000000000000 0.5441191443750029 # S**

**0.1666666664105421 0.3333333328210770 0.4505569803124985 # Cu**

**0.1666666664105421 0.3333333328210770 0.0790555821875003 # Cu**

**0.3333333335894579 0.1666666671789230 0.1677954428124977 # Cu**

**0.3333333335894579 0.1666666671789230 0.3673296353125011 # Cu**

**0.1666666664105421 0.3333333328210770 0.2509636475000008 # Cu**

**0.3333333335894579 0.1666666671789230 0.5392968409375030 # Cu**

**0.6666666664105421 0.3333333328210770 0.4505569803124985 # Cu**

**0.6666666664105421 0.3333333328210770 0.0790555821875003 # Cu**

**0.8333333335894650 0.1666666671789230 0.1677954428124977 # Cu**

**0.8333333335894650 0.1666666671789230 0.3673296353125011 # Cu**

**0.6666666664105421 0.3333333328210770 0.2509636475000008 # Cu**

**0.8333333335894650 0.1666666671789230 0.5392968409375030 # Cu**

**0.1666666671789230 0.8333333343578460 0.4505569803124985 # Cu**

**0.1666666671789230 0.8333333343578460 0.0790555821875003 # Cu**

**0.3333333335894579 0.6666666671789230 0.1677954428124977 # Cu**

**0.3333333335894579 0.6666666671789230 0.3673296353125011 # Cu**

**0.1666666671789230 0.8333333343578460 0.2509636475000008 # Cu**

**0.3333333335894579 0.6666666671789230 0.5392968409375030 # Cu**

**0.6666666671789230 0.8333333343578460 0.4505569803124985 # Cu**

**0.6666666671789230 0.8333333343578460 0.0790555821875003 # Cu**

**0.8333333335894579 0.6666666671789230 0.1677954428124977 # Cu**

**0.8333333335894579 0.6666666671789230 0.3673296353125011 # Cu**

**0.6666666671789230 0.8333333343578460 0.2509636475000008 # Cu**

**0.8333333335894579 0.6666666671789230 0.5392968409375030 # Cu**

**0.3287940658791300 0.1838840198403275 0.6164177613752031 # C**

**0.4783820525197129 0.1857150545753470 0.6304069873480884 # O**

**0.1737636260812891 0.1775385524583655 0.6380353913139700 # O**

**0.2107851624486301 0.1774943153932256 0.6675630200621612 # H**

**Title (*OCHO/CuSe)**

**1.00000000000000**

**8.0254689999999993 0.0000000000000000 0.0000000000000000**

**-4.0127344999999996 6.9502600299999999 0.0000000000000000**

**0.0000000000000000 0.0000000000000000 32.0000000000000000**

**Se Cu C O H**

**24 24 1 2 1**

**Selective dynamics**

**Direct**

**0.0000000000000000 0.0000000000000000 0.0728912859375015# Se**

**0.1666666664268703 0.3333333328537336 0.1743907053124971# Se**

**0.0000000000000000 0.0000000000000000 0.3501751659375003# Se**

**0.0000000000000000 0.0000000000000000 0.2731815900000001# Se**

**0.0000000000000000 0.0000000000000000 0.5504654696875022# Se**

**0.3333333332134316 0.1666666664268703 0.4489671628125009# Se**

**0.5000000000000000 0.0000000000000000 0.0728912859375015# Se**

**0.6666666664268703 0.3333333328537336 0.1743907053124971# Se**

**0.5000000000000000 0.0000000000000000 0.3501751659375003# Se**

**0.5000000000000000 0.0000000000000000 0.2731815900000001# Se**

**0.5000000000000000 0.0000000000000000 0.5504654696875022# Se**

**0.8333333332134316 0.1666666664268703 0.4489671628125009# Se**

**0.0000000003596980 0.5000000007193961 0.0728912859375015# Se**

**0.1666666667865684 0.8333333335731297 0.1743907053124971# Se**

**0.0000000003596980 0.5000000007193961 0.3501751659375003# Se**

**0.0000000003596980 0.5000000007193961 0.2731815900000001# Se**

**0.0000000003596980 0.5000000007193961 0.5504654696875022# Se**

**0.3333333335731297 0.6666666671462664 0.4489671628125009# Se**

**0.5000000003596980 0.5000000007193961 0.0728912859375015# Se**

**0.6666666667865684 0.8333333335731297 0.1743907053124971# Se**

**0.5000000003596980 0.5000000007193961 0.3501751659375003# Se**

**0.5000000003596980 0.5000000007193961 0.2731815900000001# Se**

**0.5000000003596980 0.5000000007193961 0.5504654696875022# Se**

**0.8333333335731297 0.6666666671462664 0.4489671628125009# Se**

**0.1666666664268703 0.3333333328537336 0.0906196746875025# Cu**

**0.1666666664268703 0.3333333328537336 0.4514632415625002# Cu**

**0.3333333332134316 0.1666666664268703 0.3720736968749989# Cu**

**0.1666666664268703 0.3333333328537336 0.2512841709375024# Cu**

**0.3333333332134316 0.1666666664268703 0.5327370809375012# Cu**

**0.3333333332134316 0.1666666664268703 0.1718946265624979# Cu**

**0.6666666664268703 0.3333333328537336 0.0906196746875025# Cu**

**0.6666666664268703 0.3333333328537336 0.4514632415625002# Cu**

**0.8333333332134316 0.1666666664268703 0.3720736968749989# Cu**

**0.6666666664268703 0.3333333328537336 0.2512841709375024# Cu**

**0.8333333332134316 0.1666666664268703 0.5327370809375012# Cu**

**0.8333333332134316 0.1666666664268703 0.1718946265624979# Cu**

**0.1666666667865684 0.8333333335731297 0.0906196746875025# Cu**

**0.1666666667865684 0.8333333335731297 0.4514632415625002# Cu**

**0.3333333335731297 0.6666666671462664 0.3720736968749989# Cu**

**0.1666666667865684 0.8333333335731297 0.2512841709375024# Cu**

**0.3333333335731297 0.6666666671462664 0.5327370809375012# Cu**

**0.3333333335731297 0.6666666671462664 0.1718946265624979# Cu**

**0.6666666667865684 0.8333333335731297 0.0906196746875025# Cu**

**0.6666666667865684 0.8333333335731297 0.4514632415625002# Cu**

**0.8333333335731297 0.6666666671462664 0.3720736968749989# Cu**

**0.6666666667865684 0.8333333335731297 0.2512841709375024# Cu**

**0.8333333335731297 0.6666666671462664 0.5327370809375012# Cu**

**0.8333333335731297 0.6666666671462664 0.1718946265624979# Cu**

**0.1408544347210920 0.1001731266320860 0.6322560270309836# C**

**0.2040114869420151 0.2686077938856002 0.6239512931658240# O**

**0.0275186498369422 0.9485101150209445 0.6081593842571138# O**

**0.1715038353127625 0.0452402217053915 0.6615343202496504# H**

**Title (*COOH/CuSe)**

**1.00000000000000**

**8.0254689999999993 0.0000000000000000 0.0000000000000000**

**-4.0127344999999996 6.9502600299999999 0.0000000000000000**

**0.0000000000000000 0.0000000000000000 32.0000000000000000**

**Se Cu C O H**

**24 24 1 2 1**

**Selective dynamics**

**Direct**

**0.0000000000000000 0.0000000000000000 0.0728912859375015# Se**

**0.1666666664268703 0.3333333328537336 0.1743907053124971# Se**

**0.0000000000000000 0.0000000000000000 0.3501751659375003# Se**

**0.0000000000000000 0.0000000000000000 0.2731815900000001# Se**

**0.0000000000000000 0.0000000000000000 0.5504654696875022# Se**

**0.3333333332134316 0.1666666664268703 0.4489671628125009# Se**

**0.5000000000000000 0.0000000000000000 0.0728912859375015# Se**

**0.6666666664268703 0.3333333328537336 0.1743907053124971# Se**

**0.5000000000000000 0.0000000000000000 0.3501751659375003# Se**

**0.5000000000000000 0.0000000000000000 0.2731815900000001# Se**

**0.5000000000000000 0.0000000000000000 0.5504654696875022# Se**

**0.8333333332134316 0.1666666664268703 0.4489671628125009# Se**

**0.0000000003596980 0.5000000007193961 0.0728912859375015# Se**

**0.1666666667865684 0.8333333335731297 0.1743907053124971# Se**

**0.0000000003596980 0.5000000007193961 0.3501751659375003# Se**

**0.0000000003596980 0.5000000007193961 0.2731815900000001# Se**

**0.0000000003596980 0.5000000007193961 0.5504654696875022# Se**

**0.3333333335731297 0.6666666671462664 0.4489671628125009# Se**

**0.5000000003596980 0.5000000007193961 0.0728912859375015# Se**

**0.6666666667865684 0.8333333335731297 0.1743907053124971# Se**

**0.5000000003596980 0.5000000007193961 0.3501751659375003# Se**

**0.5000000003596980 0.5000000007193961 0.2731815900000001# Se**

**0.5000000003596980 0.5000000007193961 0.5504654696875022# Se**

**0.8333333335731297 0.6666666671462664 0.4489671628125009# Se**

**0.1666666664268703 0.3333333328537336 0.0906196746875025# Cu**

**0.1666666664268703 0.3333333328537336 0.4514632415625002# Cu**

**0.3333333332134316 0.1666666664268703 0.3720736968749989# Cu**

**0.1666666664268703 0.3333333328537336 0.2512841709375024# Cu**

**0.3333333332134316 0.1666666664268703 0.5327370809375012# Cu**

**0.3333333332134316 0.1666666664268703 0.1718946265624979# Cu**

**0.6666666664268703 0.3333333328537336 0.0906196746875025# Cu**

**0.6666666664268703 0.3333333328537336 0.4514632415625002# Cu**

**0.8333333332134316 0.1666666664268703 0.3720736968749989# Cu**

**0.6666666664268703 0.3333333328537336 0.2512841709375024# Cu**

**0.8333333332134316 0.1666666664268703 0.5327370809375012# Cu**

**0.8333333332134316 0.1666666664268703 0.1718946265624979# Cu**

**0.1666666667865684 0.8333333335731297 0.0906196746875025# Cu**

**0.1666666667865684 0.8333333335731297 0.4514632415625002# Cu**

**0.3333333335731297 0.6666666671462664 0.3720736968749989# Cu**

**0.1666666667865684 0.8333333335731297 0.2512841709375024# Cu**

**0.3333333335731297 0.6666666671462664 0.5327370809375012# Cu**

**0.3333333335731297 0.6666666671462664 0.1718946265624979# Cu**

**0.6666666667865684 0.8333333335731297 0.0906196746875025# Cu**

**0.6666666667865684 0.8333333335731297 0.4514632415625002# Cu**

**0.8333333335731297 0.6666666671462664 0.3720736968749989# Cu**

**0.6666666667865684 0.8333333335731297 0.2512841709375024# Cu**

**0.8333333335731297 0.6666666671462664 0.5327370809375012# Cu**

**0.8333333335731297 0.6666666671462664 0.1718946265624979# Cu**

**0.3307703257653500 0.6565110244552628 0.6465858795346477# C**

**0.4767189325695469 0.6736622953291729 0.6612933859754264# O**

**0.1684809592466294 0.6125478814018877 0.6665590378664277# O**

**0.1935499489074815 0.5966361096144084 0.6962255167326390# H**

**Title (*OCHO/CuTe)**

**1.00000000000000**

**6.3099999999999996 0.0000000000000000 0.0000000000000000**

**0.0000000000000000 8.1839999999999993 0.0000000000000000**

**0.0000000000000000 0.0000000000000000 27.8239990000000006**

**Te Cu C O H**

**12 16 1 2 1**

**Selective dynamics**

**Direct**

**0.1250000000000000 0.1250000000000000 0.0912650000454676# Te**

**0.3750000000000000 0.3750000000000000 0.2306150000221052# Te**

**0.6250000000000000 0.1250000000000000 0.0912650000454676# Te**

**0.8750000000000000 0.3750000000000000 0.2306150000221052# Te**

**0.1250000000000000 0.6250000000000000 0.0912650000454676# Te**

**0.3750000000000000 0.8750000000000000 0.2306150000221052# Te**

**0.6250000000000000 0.6250000000000000 0.0912650000454676# Te**

**0.8750000000000000 0.8750000000000000 0.2306150000221052# Te**

**0.1250000000000000 0.1250000000000000 0.3412650000454676# Te**

**0.6250000000000000 0.1250000000000000 0.3412650000454676# Te**

**0.1250000000000000 0.6250000000000000 0.3412650000454676# Te**

**0.6250000000000000 0.6250000000000000 0.3412650000454676# Te**

**0.1250000000000000 0.3750000000000000 0.1491149999681909# Cu**

**0.3750000000000000 0.1250000000000000 0.1727650000993748# Cu**

**0.6250000000000000 0.3750000000000000 0.1491149999681909# Cu**

**0.8750000000000000 0.1250000000000000 0.1727650000993748# Cu**

**0.1250000000000000 0.8750000000000000 0.1491149999681909# Cu**

**0.3750000000000000 0.6250000000000000 0.1727650000993748# Cu**

**0.6250000000000000 0.8750000000000000 0.1491149999681909# Cu**

**0.8750000000000000 0.6250000000000000 0.1727650000993748# Cu**

**0.1250000000000000 0.3750000000000000 0.3991149999681909# Cu**

**0.3750000000000000 0.1250000000000000 0.4227650000993748# Cu**

**0.6250000000000000 0.3750000000000000 0.3991149999681909# Cu**

**0.8750000000000000 0.1250000000000000 0.4227650000993748# Cu**

**0.1250000000000000 0.8750000000000000 0.3991149999681909# Cu**

**0.3750000000000000 0.6250000000000000 0.4227650000993748# Cu**

**0.6250000000000000 0.8750000000000000 0.3991149999681909# Cu**

**0.8750000000000000 0.6250000000000000 0.4227650000993748# Cu**

**0.3791330867181912 0.6328492977727009 0.5235893832473835# C**

**0.2568367710811330 0.6255283257936242 0.4854417261078865# O**

**0.5743827271002644 0.6397019593524789 0.5247594891279874# O**

**0.2818987533299122 0.6320230668283742 0.5569446737361758# H**

**Title (*COOH/CuTe)**

**1.00000000000000**

**6.3099999999999996 0.0000000000000000 0.0000000000000000**

**0.0000000000000000 8.1839999999999993 0.0000000000000000**

**0.0000000000000000 0.0000000000000000 27.8240000000000016**

**Te Cu C O H**

**12 16 1 2 1**

**Selective dynamics**

**Direct**

**0.1250000000000000 0.1250000000000000 0.0912649967653820# Te**

**0.3750000000000000 0.3750000000000000 0.2306149917337521# Te**

**0.6250000000000000 0.1250000000000000 0.0912649967653820# Te**

**0.8750000000000000 0.3750000000000000 0.2306149917337521# Te**

**0.1250000000000000 0.6250000000000000 0.0912649967653820# Te**

**0.3750000000000000 0.8750000000000000 0.2306149917337521# Te**

**0.6250000000000000 0.6250000000000000 0.0912649967653820# Te**

**0.8750000000000000 0.8750000000000000 0.2306149917337521# Te**

**0.1250000000000000 0.1250000000000000 0.3412649877803346# Te**

**0.6250000000000000 0.1250000000000000 0.3412649877803346# Te**

**0.1250000000000000 0.6250000000000000 0.3412649877803346# Te**

**0.6250000000000000 0.6250000000000000 0.3412649877803346# Te**

**0.1250000000000000 0.3750000000000000 0.1491149946089720# Cu**

**0.3750000000000000 0.1250000000000000 0.1727649938901692# Cu**

**0.6250000000000000 0.3750000000000000 0.1491149946089720# Cu**

**0.8750000000000000 0.1250000000000000 0.1727649938901692# Cu**

**0.1250000000000000 0.8750000000000000 0.1491149946089720# Cu**

**0.3750000000000000 0.6250000000000000 0.1727649938901692# Cu**

**0.6250000000000000 0.8750000000000000 0.1491149946089720# Cu**

**0.8750000000000000 0.6250000000000000 0.1727649938901692# Cu**

**0.1250000000000000 0.3750000000000000 0.3991149856239247# Cu**

**0.3750000000000000 0.1250000000000000 0.4227649849051147# Cu**

**0.6250000000000000 0.3750000000000000 0.3991149856239247# Cu**

**0.8750000000000000 0.1250000000000000 0.4227649849051147# Cu**

**0.1250000000000000 0.8750000000000000 0.3991149856239247# Cu**

**0.3750000000000000 0.6250000000000000 0.4227649849051147# Cu**

**0.6250000000000000 0.8750000000000000 0.3991149856239247# Cu**

**0.8750000000000000 0.6250000000000000 0.4227649849051147# Cu**

**0.3893620036483654 0.6309084247847210 0.4920635579343298# C**

**0.4638150302916770 0.5187200578706310 0.5158205798689082# O**

**0.3160609411478816 0.7691749119279692 0.5142768294529674# O**

**0.3407103256122141 0.7519978827501745 0.5490306405278744# H**
